# A Multiscale Approach Indicates a Severe Reduction in Atlantic Forest Wetlands and Highlights that São Paulo Marsh Antwren Is on the Brink of Extinction

**DOI:** 10.1371/journal.pone.0121315

**Published:** 2015-03-23

**Authors:** Glaucia Del-Rio, Marco Antonio Rêgo, Luís Fábio Silveira

**Affiliations:** 1 Departamento de Zoologia, Instituto de Biociências, Universidade de São Paulo, São Paulo, São Paulo, Brazil; 2 Seção de Aves, Museu de Zoologia da Universidade de São Paulo, São Paulo, São Paulo, Brazil; Institute of Agronomy, University of Lisbon, PORTUGAL

## Abstract

Over the last 200 years the wetlands of the Upper Tietê and Upper Paraíba do Sul basins, in the southeastern Atlantic Forest, Brazil, have been almost-completely transformed by urbanization, agriculture and mining. Endemic to these river basins, the São Paulo Marsh Antwren (*Formicivora paludicola*) survived these impacts, but remained unknown to science until its discovery in 2005. Its population status was cause for immediate concern. In order to understand the factors imperiling the species, and provide guidelines for its conservation, we investigated both the species’ distribution and the distribution of areas of suitable habitat using a multiscale approach encompassing species distribution modeling, fieldwork surveys and occupancy models. Of six species distribution models methods used (Generalized Linear Models, Generalized Additive Models, Multivariate Adaptive Regression Splines, Classification Tree Analysis, Artificial Neural Networks and Random Forest), Random Forest showed the best fit and was utilized to guide field validation. After surveying 59 sites, our results indicated that *Formicivora paludicola* occurred in only 13 sites, having narrow habitat specificity, and restricted habitat availability. Additionally, historic maps, distribution models and satellite imagery showed that human occupation has resulted in a loss of more than 346 km2 of suitable habitat for this species since the early twentieth century, so that it now only occupies a severely fragmented area (area of occupancy) of 1.42 km2, and it should be considered Critically Endangered according to IUCN criteria. Furthermore, averaged occupancy models showed that marshes with lower cattail (*Typha dominguensis*) densities have higher probabilities of being occupied. Thus, these areas should be prioritized in future conservation efforts to protect the species, and to restore a portion of Atlantic Forest wetlands, in times of unprecedented regional water supply problems.

## Introduction

Wetlands have always attracted humans, especially for occupation, due principally to their fertile soils and high productivity, with many of the great historic civilizations having arisen in and near river floodplains [1]. They are one of the most productive habitats on Earth, supporting many kinds of life [1]. Their food web is composed largely of invertebrates that feed on decaying plants, with vertebrates such as fish, reptiles, mammals and birds occupying the highest trophic levels [1].

Within the São Paulo metropolitan region, rivers and wetlands used to comprise a substantial portion of the landscape [2]. Reports and historic maps show that within the city limits alone, there were once more than 80 km of wetlands extending along the Tietê, Pinheiros and Tamanduateí Rivers [2]. According to Jorge [2], these areas used to shelter brocket deer, otters, rails and many types of fish. Today, this region has one of the highest human concentrations in the world, with almost 20 million people [3]. Development has generally been disorderly, and its environmental impacts are numerous [2]. These wetlands are part of a mosaic of different landscape formations with distinct ecological borders that together form the Atlantic Forest domain: a principal biodiversity hotspot, and one of the most endangered domains in the world [4], [5]. This is the result of a history of intense exploitation involving wood extraction, mining, agriculture, industrialization and urbanization [6], [7], [8], [9]. However, of all the formations, wetlands are one of the most neglected and least studied and their biodiversity is now in great danger [2].

The São Paulo Marsh Antwren (*Formicivora paludicola*) (Thamnophilidae) [10] is an example of this. It is a passerine endemic to the wetlands of the São Paulo state, yet it remained unknown to science until its discovery in the early twenty-first century. It is the only bird species and one of the few vertebrates endemic to this region [11]. *Formicivora paludicola* occurs exclusively in wetlands, especially those dominated by cattail (*Typha dominguensis*). Thamnophilids are not attractive for hunting or for pet trade, and the main threat facing *F*. *paludicola* is habitat destruction [12].

Today, the species only occurs in marshes in the Upper Tietê and Upper Paraíba do Sul basins. The localities are few and sparsely distributed. In general, natural areas in this region are few and disconnected. For wetlands, the situation is even worse, considering their delicate dynamics and neglect by authorities and lay population. Nevertheless, there are still some wetlands that have not yet been urbanized although they all are encircled by pastures, mining, plantations and industrial activities [2], [13]. The conservation status of *F*. *paludicola* is therefore of concern. However, due to its recent discovery almost every aspect of its natural history has not been studied, including its distribution [14].

Predicting a species’ distribution plays a significant component in planning conservation efforts, and has other important applications in ecology [15], [16], [17], [18], [19], [20], [21]. The main objective is to convert point information of a species’ distribution into predictive maps, using climatic, topographic, and edaphic data [22]. For species with records from only a few localities, Species Distribution Models (SDMs) may be useful for providing a description of areas climatically similar to the ones in which the species is known to occur, helping to improve field surveys [23], [24]. Furthermore, ecologists recognize the necessity of focusing conservation efforts on species with higher risks of population decline, and have worked to identify priority groups, habitats, and areas that are in greatest need of conservation [25], [26], [27]. Such prioritization should be based on environmental features that benefit the maintenance of biodiversity. Occupancy models can then be used with the intent of relating species presence/absence to the characteristics of the sampling locations [28].

Integrating distribution models (i.e. species-climate envelope models), field surveys and occupancy models, we applied a novel multiscale approach, that could be applied for threatened species worldwide, to assess the threat profile for a species and of its environment. With a compilation of systematized information for this species, its habitat requirements, and the status of São Paulo’s wetlands, we define the prioritization strategies for conservation activities in these Atlantic Forest Wetlands.

## Methods

### Distribution Models

In order to find climatic suitable areas for the species along its biome, we generated distribution models and predictive maps using all 15 known localities (15 marshes) of *F*. *paludicola* (S1 Table). Climate and altitude data for each location were extracted from the WorldClim database (Tile 34—encompassing most of Atlantic Forest latitudes) [29] at a resolution of 30 arc-seconds (approximately 1 km). For eliminating spatially correlated variables (from all 19 climate variables from WorldClim database) prior to the modeling process, we used the “Correlation” tool in ENM Tools 1.3 software [30]. We analyzed the Pearson correlation coefficient for every pair-wise comparison of raster files, and retained the five least correlated climate variables (Pearson’s < 0.7). The variables used were: isothermality (mean diurnal range/temperature annual range), temperature seasonality, temperature annual range, precipitation of driest month and precipitation seasonality. We also used two variables related to hydrology: height above the nearest drainage channel [31] and drainage channel density (obtained through SRTM: Shuttle Radar Topographic Mission images from the HydroSHEDS project [32], with a resolution of 1 km [33]). Both these were obtained from the Ambdata INPE [34].

Using the BIOMOD [35] framework for the R programming environment [36], we applied six methods to detect the model with the highest accuracy: Generalized Linear Models (GLMs) [37], Generalized Additive Models (GAMs) [38], Multivariate Adaptive Regression Splines (MARS) [39], Classification Tree Analysis (CTA) [40], Artificial Neural Networks (ANN) [41] and Random Forest (RF) [42]. These methods are at the forefront of species distribution modeling [22], [43], [44], [45] and some of them have been shown to be very accurate in studies which dealt with species with localized distribution [46], [47]. For further information on the procedures used to run each model see: Thuiller [35] (GLM, GAM, CTA, ANN), Muñoz and Felicísimo [48] (MARS), Breiman and Cutler [49] (RF), S2 Table and S1 Appendix. We chose the number of Pseudo-Absences and the method of Pseudo-Absences selection following guidelines proposed by Barbet-Massin et al. [50]: 10,000 Pseudo-Absences in GLM, GAM, MARS and ANN; and 15 (the same number as presences) in CTA and RF.

The original models were reclassified into binary models of presence/absence using a threshold approach. Because only presence data were available we used a method that maximizes sensitivity and specificity to calculate the threshold levels [51]. Model predictive performance was evaluated using true skills statistic (TSS) [52], area under the curve (AUC) of receiver operating characteristic (ROC) [53], and Cohen’s kappa statistic [54]. TSS ranges from-1 to +1, where +1 indicates perfect agreement and values of zero or less indicate a performance no better than random [52]. Regarding AUC, values > 0.8 indicate high model accuracy [55]. Kappa values range from-1 to +1, where values near +1 indicate high performance [54]. We used the best model to generate the predictive map which guided our field surveys.

### Validation

**Fig 1:**
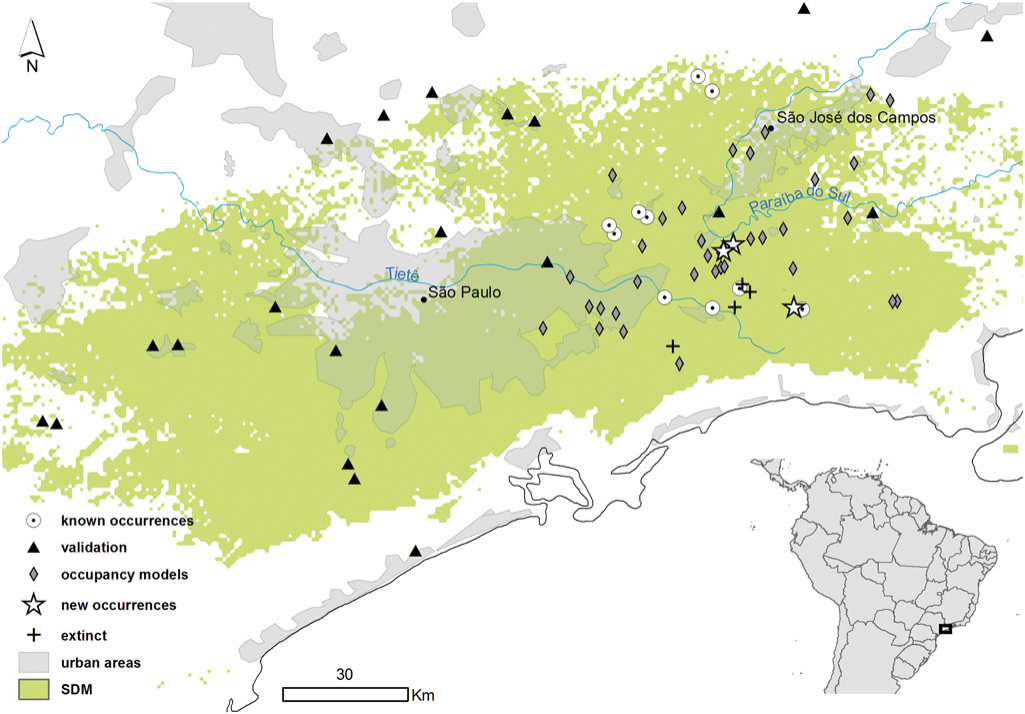
Species Distribution Model results and surveyed areas. Green areas represent Random Forest projections of areas climatically suitable for São Paulo Marsh Antwren (*Formicivora paludicola*). Grey shading represents urban areas. Circles represent areas known to possess *F*. *paludicola* that were used in occupancy models. Triangles represent areas visited for the distribution model validation where the species is absent. Diamonds represent areas visited three times for occupancy models surveys where the species is absent. Stars represent new areas discovered to possess *F*. *paludicola* that were used in the occupancy models. Crosses represent areas where the species could be extinct. Some validation localities are not shown on the map.

For the SDM validation we searched for wetlands within and adjacent to the limits indicated by the best SDM (Fig. 1). We visually scanned satellite images looking for marshes according to their color patterns. To highlight wetlands in these images, we used bands from the OLI (Operational Land Imager) sensor onboard LANDSAT 8 [56]. Using ArcGIS Desktop 10.1, we performed a false color classification (6, 5, 4—RGB). To obtain a spatial resolution of 15 m, we applied a fusion technique with the panchromatic band (band 8).

We selected 57 areas in 32 different municipalities in São Paulo State and two in Rio de Janeiro State (S3 Table). We visited these areas at least twice over the two years from October 2011 to October 2013. Knowing the territorial behavior of the species, we used three playback sessions at each visit to increase its detection probability. Each session included one minute of playback (loudsong and calls of males and females) separated by five minutes of silence (protocol modified from Boscolo et al. [57]).

### Distribution Range Size

In order to investigate the conservation status of the species, we calculated its extent of occurrence (EOO) using the minimum convex polygon (MCP) approach [58]. We calculated the suitable area predicted by the SDM within the MCP (Environmental Suitability in Extent of Occurrence ES-EOO) [24]. Additionally, we measured the area of occupancy (AOO) by counting the number of occupied cells in a grid of 330 m X 330 m covering the entire distribution (according to the average areas occupied size—0.11 km^2^), and estimating the total area of occupied cells [58].

### Occupancy Models

Occupancy models allow inference about the distribution of organisms over space, using temporal and spatial replication to produce estimates of occupancy and detection probabilities, and their related parameters [59]. We used these models to investigate the manner in which the probability of occupancy of *F*. *paludicola* varies with the characteristics of the sites. In a more detailed scale than species distribution models, occupancy models were built focused on marshes, to find suitable conditions for the local occurrence of the species, thus testing the effect of several environmental covariates on the occupancy probabilities of the São Paulo Marsh Antwren. To create the occupancy models we used a total of 47 areas. Of the 15 localities where *F*. *paludicola* was known to occur (used in the SDM), we included only nine as five wetlands no longer existed due to human activities and one was under direct influence of mining activities (Fig. 1). To these we added a further 38 marshes that fell within the MCP of the São Paulo Marsh Antwren distribution with a buffer of 30 km [60] that had previously been used in the SDM validation. We systematically visited each area three times, with an interval of _~_15 days between visits from August to September 2013. We alternated the time of the visits (from 6h A.M. to 11h A.M.), and performed the playback routine described above on every visit. When at least one individual was detected on the first visit, we made positive detections on the next two visits to the same site. Therefore, we considered the detection probability to be constant among visits.

The selected variables were wetland size (m^2^), presence of native forest matrix adjacent to the marshes, presence of commercial plantations (such as *Eucalyptus*), presence of mining activities, distance to main highways and roads, distance to urban areas, distance to rivers, and water flow (maximum, average and minimum). We created vectors delimiting the marshes, urban areas and paved roads based on a visual classification of BirdsEye Satellite Imagery in Base Camp, obtaining patch size and minimum distance from the centre of the marshes to urban areas borders and to roads in ArcGIS 10.1. Visually scanning the same images, we were able to indicate presence and absence of *Eucalyptus* plantation, mining activities and native forest matrix adjacent to each marsh. In order to obtain hydrological data, we used the Hydrology toolset in Spatial Analyst extension of ArcGIS 10.1 [61]. Based on ASTER GDEM 2 imagery [62], we calculated the water flow direction to obtain the flow accumulation raster and the drainage network. With the delineated drainage network it was possible to calculate the minimum distance between marsh centers and water courses displayed in the imagery. We also used average cattail *Typha dominguensis* density and height as covariates in the models. These were collected in the field, at each sample site, by counting cattail stalks in three 1 m^2^ quadrats, randomly distributed in the sample site, for density estimates and then using the sward stick method for vegetal height estimates [63], [64], [65]. These features were assumed to be homogeneous within each site. The choice of these variables was based on their ability to reflect the intensity of human occupation, the age of the marsh and natural features, i.e. they were used to investigate how human activities and environmental traits might influence *F*. *paludicola* occupancy probabilities.

We used occupancy models to estimate the probability of the species occurring at a site (ψ). Detection histories were built for each site [59] (S4 Table and S1 Dataset), and site-specific covariates were converted to standard normalized z scores. Our design corresponds to a “single-species, single-season occupancy model”, assuming that we had a closed population over the short period that the visits took place (54 days) [59]. This modeling could be considered a form of generalized logistic regression analysis as there is some uncertainty as to whether an observed absence equates to a true absence [59]. While the probability of occupancy varies among sites, the parameters being estimated are the β values, or the regression coefficients of each covariate [59]. We built models using the package “unmarked” in the R software [66] (S2 Appendix), and began by fitting candidate single-season models, allowing them to vary with individual or additively combined site-specific covariates. In order to rank candidate models, we used the Akaike Information Criterion (AIC) [67], [68] by calculating the difference between the best model (with the lowest AIC value) and all other models (ΔAIC) as well as using Akaike weights (ωi) (i. e., the conditional probability for each model). We calculated the strength of evidence in favor of one model over another by dividing their Akaike weights to obtain the evidence ratio [69]. We excluded models from our analysis that did not converge. We also examined the confidence intervals for each of the estimated parameters (95%), excluding models with highly inclusive confidence intervals (with negative, zero and positive values). We applied model averaging method to estimate occupancy probability from the multiple models tested [70], we also calculated the relative importance of the model parameters in R package AICmodavg [71].

Field work was undertaken in accordance with Brazilian laws (permit number: 31224 Ministério do Meio Ambiente/ Instituto Chico Mendes de Conservação da Biodiversidade/ Sistema de Autorização e Informação em Biodiversidade). Areas accessed were privately owned and we did not sample protected species. The owners of the lands (Suzano Papel e Celulose, and small agricultural producers) gave permission to conduct the study on each site.

## Results

### Distribution Model and Distribution Range Size

Because of a slight advantage over GAM and GLM, we used the RF (AUC = 1, Kappa = 1 and TSS = 1) results to direct the field work procedure. The RF model showed habitat suitability in a very restricted area around the known distribution localities, and predicted a small westward extension into São Paulo state (Fig. 1). The analysis of variable contribution showed that isothermality had a relative contribution of 32% to the model; temperature seasonality, 29%; altitude, 25%; and annual temperature range, 14%.

**Fig 2:**
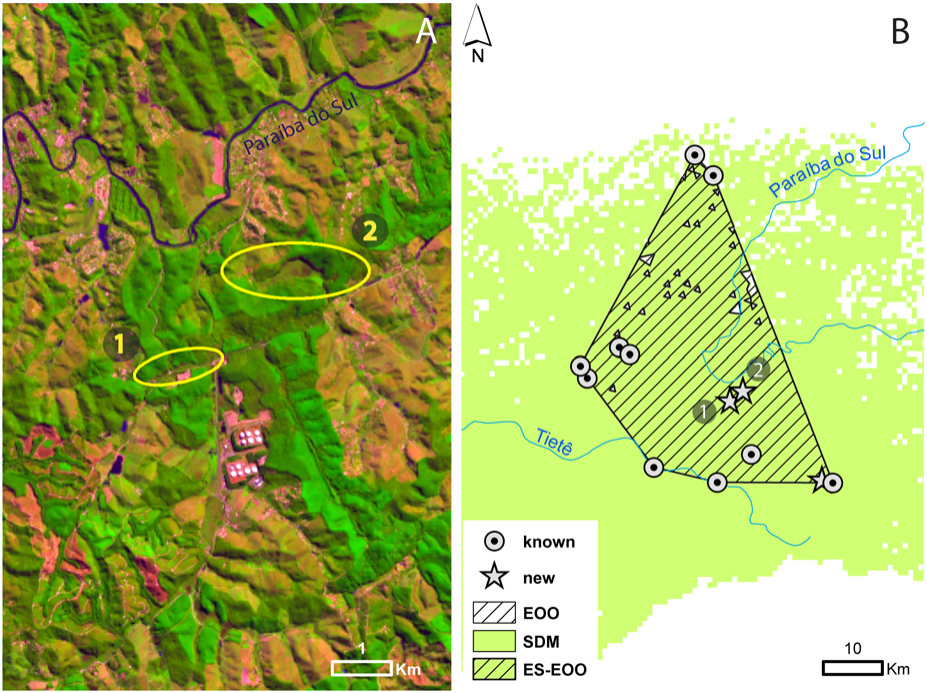
Occurrence of *Formicivora paludicola*. A) Example of LANDSAT 8 satellite image visually scanned in the search for marshes to be surveyed. Numbers 1 and 2 indicate new areas of occurrence *Formicivora paludicola*. B) Circles represent areas of previously known occurrence that still possess the species; Stars represent new areas of occurrence discovered during our field surveys; Numbers 1 and 2 indicate the same areas of A; Green areas represent Random Forest model projections of areas climatically suitable for *F*. *paludicola*. The EOO (extent of occurrence) was estimated at 1,268 km^2^ and ES-EOO (environmental suitability in extent of occurrence) at 1,245 km^2^.

Of the 57 areas surveyed, *F*. *paludicola* was not detected in 54 of the areas, and found in only three new areas. These three sites were predicted to be part of the potential distribution of the species by the outcome of the model. We registered 42 points of absence in areas where the presence had been predicted, resulting in a commission error of 93%. Another 17 true absences were recorded in areas in which *F*. *paludicola* was predicted to be absent (Fig. 1). The EOO was estimated at 1,268 km^2^, the ES-EOO at 1,245 km^2^ (Fig. 2) and AOO at 1.42 km^2^.

### Occupancy Models

**Fig 3:**
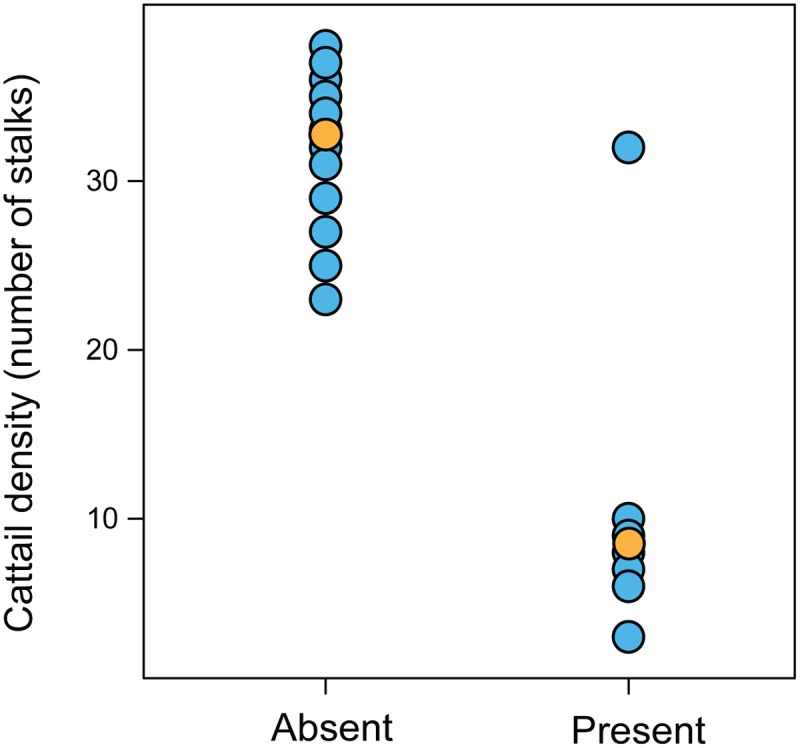
Differences between density of cattail (*Typha dominguensis*) in sites where *Formicivora paludicola* is absent or present. Orange dots represent average values.

The São Paulo Marsh Antwren was detected in 12 of the 47 selected areas. Model Averaging using all the models that converged (58 of which 13 are displayed in Table 1, the others are in S5 Table) and had reliable confidence intervals showed that the main covariable affecting negatively the probability of occupancy of *F*. *paludicola* was *Typha dominguensis* density (β_Typhadensity_ = -3.75 ±1.42 SE) (Table 2; Fig. 3). The probability of occupancy was not affected by other variables such as *Typha dominguensis* height (β_Typhaheight_ = 1.3 ±1.54 SE); distance to highways (β_Highways_ = 0.76 ±0.87 SE); and presence of *Eucalyptus* plantations adjacent to the marsh (β_Eucalyptus_ = -1.33 ±2.36 SE) (Table 2). Model averaged prediction of occupancy was 0.25 ± 0.04 SE (CI_2.5–97.5_ = 0.09–0.46), a value slightly higher than the proportion of sites where *F*. *paludicola* was recorded (0.23), although this falls within the confidence interval for the estimates.

**Table 1:** 13 best models tested for probabilities of occupancy of *Formicivora paludicola*.

Models	N. parameters	AICc	ΔAICc	AICc weight ωi
Ψ(Typha density)p(.)	3	17.26	0	0.22
Ψ(Typha density+Typha height)p(.)	4	18.64	1.38	0.11
Ψ(Typha density+Distance to highway)p(.)	4	19.01	1.75	0.09
Ψ(Typha density+Eucalyptus plantation)p(.)	4	19.2	1.94	0.08
Ψ(Typha density+Maximum water flow)p(.)	4	19.42	2.16	0.07
Ψ(Typha density+Distance to city)p(.)	4	19.44	2.18	0.07
Ψ(Typha density+Mining)p(.)	4	19.46	2.2	0.07
Ψ(Typha density+Distance to Rivers)p(.)	4	19.56	2.3	0.07
Ψ(Typha density+Marsh area)p(.)	4	19.58	2.32	0.07
Ψ(Typha density+Typha height+Distance to highway)p(.)	5	20.14	2.88	0.05
Ψ(Typha density+Average water flow)p(.)	4	19.65	2.39	0.07
Ψ(Typha density+Typha height+Distance to highway+Eucalyptus plantation)p(.)	6	22.61	5.35	0.02
Ψ(Typha density+Typha height+Distance to highway+Eucalyptus plantation+Maximum water flow)p(.)	7	24.38	7.12	0.01

N. parameters = number of parameters estimated by the model; AICc = Akaike Information Criterion value with correction for small samples; ΔAICc = difference between each model and the best model; AICc weight = Akaike weights (ωi) (the conditional probability for each model).

**Table 2:** Averaged estimates of βs for models covariates.

Habitat variables	β	SE	CI 0.025	CI 0.975
Typha density	-3.75	1.42	-6.53	-0.97
Typha height	1.3	1.54	-1.73	4.32
Distance to highway	0.76	0.87	-0.93	2.46
Eucalyptus plantation	1.33	2.36	-5.95	3.29
Maximum water flow	1.52	3.42	-5.18	8.21
Distance to city	0.43	0.88	-1.29	2.16
Mining	-1.13	2.73	-6.49	4.23
Distance to rivers	0.26	0.73	-1.18	1.7
Marsh area	0.3	1.08	-1.81	2.41
Average water flow	0.08	1.03	-1.94	2.1

The values indicate the relationship between variables and the probability of occupancy of *Formicivora paludicola*.

## Discussion

### Distribution

The SDM had a high accuracy and indicated a very restricted area of suitable habitat for *F*. *paludicola*. This is consistent with Jiménez-Valverde et al. [72] who suggested that modelling the distribution of species that are habitat specialists is more accurate than modelling the distribution of generalists [73], [74], [75]. On the other hand, this could also be the result of the small sample size (15 sites), or the narrow range of occurrence points [76], [77]. However, the validation process showed that the real distribution could be even smaller than the potential one (commission error of 93%). We could not find the species outside the polygon that represents its current known distribution (Fig. 2). Based on the validation data, our results indicate that the distribution of *F*. *paludicola* extends only between the upper Tietê River in Salesópolis, Biritiba-Mirim and Mogi das Cruzes and the upper Paraíba do Sul River in Guararema and São José dos Campos.

Within this area, there are only a few marshes occupied by the species. We were able to record *F*. *paludicola* in only 10 of the 15 areas previously known to have been occupied [10], and in only three new areas (Figs. 1 and 2). Therefore, some of the localities that were used to run the SDMs no longer possessed the species, and at these it is probably locally extinct. Four of these areas were lost due to the construction of the Paraitinga Reservoir. Between 2006 and 2007 there was an effort to translocate individuals from these populations to new areas but the success of this is still being studied by LFS. Another area is now covered by secondary growth (Ponte Nova Dam), and another still houses the species but belongs to a mining corporation and is currently being drained (Casa Grande Road).

As mentioned above, we found only three additional localities with *F*. *paludicola* (Figs. 1 and 2), and we suggest that the species’ distribution could be considered sparse and geographically restricted to a very specific habitat. Such features often characterize rare species. Additionally, the marshes inhabited by *F*. *paludicola* are on average 4.5 km apart, while long-term studies of the dispersal ability of its sister species (*F*. *acutirostris*) show that the maximum distance traveled by a bird in its entire life was of 180 m (from the place it was born to the site where it established its territory) [78]. In the future, genetic studies could also indicate whether the populations are geographically isolated. Such isolation could be due to the presence of urban and rural development in the 30 km separating both populations, or could be generated by natural processes.

### Human occupation

**Fig 4:**
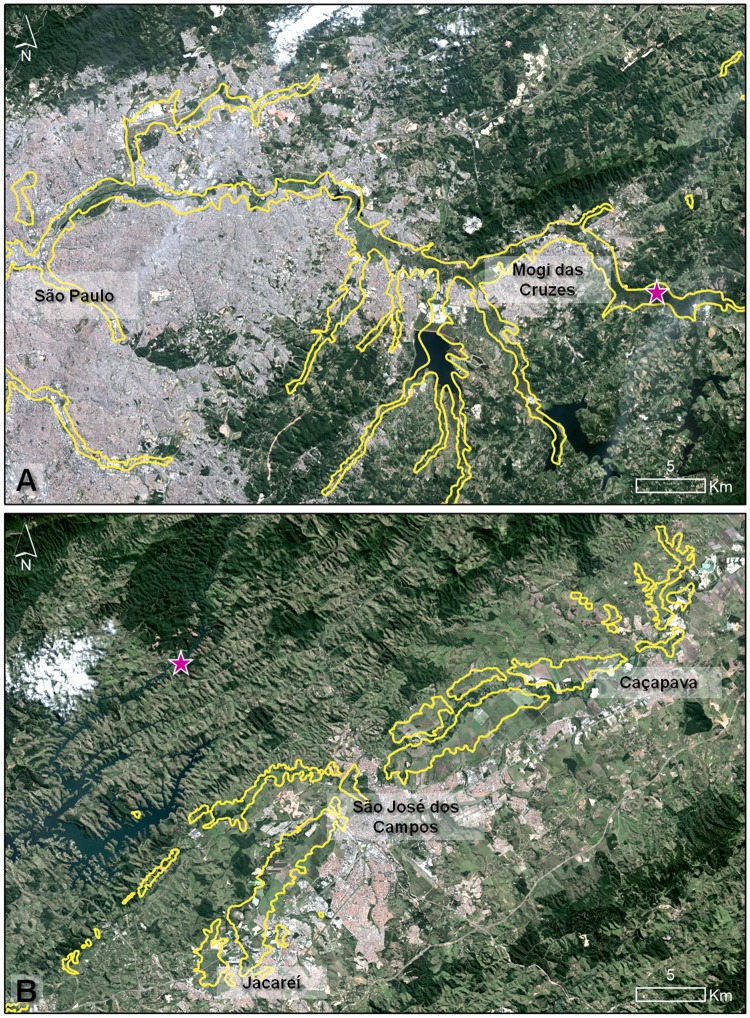
The severe reduction of São Paulo’s wetlands. Present (2013) LANDSAT images of São Paulo State superimposed on the yellow outline that represents the extent of wetlands and marshes before urbanization, in 1905, according to the Comissão Geológica e Geográfica de São Paulo. This shows the extent of human occupation of wetland areas, which have been predominantly replaced by agriculture, urbanization and mining: A) Tietê and B) Paraíba do Sul Basins in the São Paulo Metropolitan Region. Pink stars represent marshes where *Formicivora paludicola* is known to occur.

The São Paulo Marsh Antwren has experienced a severe loss of habitat in the last 100 years. Documents and maps from 1889 to 1905 show that in the urban region of São Paulo and between Jacareí and Caçapava in the Upper Paraíba do Sul Basin, there were more than 410 km^2^ of marshes and wetlands [2], [13], [79]. Current LANDSAT imagery shows that this has been almost completely replaced by mining, agriculture, pastures, industries, and urban development (Fig. 4). According to results of the Random Forest SDM, 346 out these 410 km^2^ represented climatically suitable areas for the species. This data indicate that *F*. *paludicola* could have lost more than 90% of suitable areas. New searches for suitable natural marshes and *F*. *paludicola* should therefore be done using this historical information.

### Status

Using the occurrence data, we defined the IUCN Red List category in which *F*. *paludicola* should be considered. After calculating the EOO and ES-EOO, we observed that the potential geographic distribution by Random Forest could indicate unsuitable areas for the species inside the EOO (Fig. 2). This result indicates that the extent of occurrence may be even smaller than 1,268 km^2^ (ES-EOO—1 245 km^2^). Although this EOO could be considered large when compared to that of other threatened Brazilian Thamnophilids, such as: *Formicivora littoralis* (Edangered—EOO: 260 km^2^) [80], *Formicivora erythronotos* (Endangered—EOO: 130 km^2^) [81], and *Myrmotherula snowi* (Critically Endangered—EOO: 150 km^2^) [82], its AOO is 1.42 km^2^, almost 10 times smaller than the inferior threshold for considering a species as Critically Endangered (IUCN criteria B2) [61]. This, along with the severe fragmentation of the occupied area (IUCN B2a), its limited dispersal ability, narrow habitat specificity, limited habitat availability, and possible severe population reduction over the last 100 years due to habitat loss (IUCN A2; B2c), show that *F*. *paludicola* should be classified as Critically Endangered according to IUCN Red List criteria [58].

### Enhancing Protection

Today, the Upper Tietê and Upper Paraíba do Sul basins are together occupied by around 16 million people, around 8% of entire Brazilian population [3]. This huge human presence is reflected in complex and intensive forms of land transformation and of natural resources use [83]. Marshes are highly vulnerable to the expansion of areas dedicated to agriculture, livestock farming, mining or urbanization. This has resulted in the extensive loss of wetlands in many countries throughout the world [84]. In North America, similar problems have led to a sharp decline of many wetland bird species [85], [86], [87], making these wetlands a primary conservation focus [88]. Many other bird species inhabiting the São Paulo wetlands are also considered threatened in regional Red Lists, such as the Lesser Grass-Finch (*Emberizoides ypiranganus*), Long-winged Harrier (*Circus buffoni*), and the Sickle-winged Nightjar (*Hydropsalis anomala*) [14]. Additionally there are several fish species, including *Hyphessobrycon duragenys*, *Hyphessobrycon flammeus*, *Spintherobolus papilliferus*, *Heptapterus multiradiatus*, *Pseudotocinclus parahybae*, and an amphibian *Cycloramphus semipalmatus* that are endemic to the Upper Tietê and/or Upper Paraíba do Sul Basins, and are threatened according to regional lists [89], [90]. If transformation continues at the present rate, São Paulo’s wetlands are destined to be totally destroyed or completely degraded, which would likely result in the loss of their specialist species, including *F*. *paludicola*.

Urgent conservation action is required not only to protect the target species of this project—which could be also considered a flagship species—but the complete biota of São Paulo’s wetlands. Entire ecological communities are threatened by the disorderly development that continues to transform marshes into urban areas, pastures and mines. This happens because marshes were historically considered areas of lower economical and anthropological value. However, intact wetlands are of enormous economic value due to their ability to store floodwaters, protect shorelines, improve water quality, recharge groundwater aquifers, and process nutrients [1], [91], [92], [93]. Therefore, we strongly advise the creation of protected areas and the restoration of marshes in the Upper Tietê and Paraíba do Sul basins. We also suggest the creation of a protected area, which would include the Mogi Marsh, near the Rio Acima Road, since it is the largest area which shelters *F*. *paludicola*. An even better scenario would be the creation of a reserve from Mogi das Cruzes to Salesópolis with protected and connected marshes separated by a proper buffer zone between it and the surrounding agriculture and mining activities, to reduce the contamination of their waters. The protection of a diverse spectrum of wetland types would also mitigate the problem that marshes are often transitory in nature.

### Habitat Features and Conservation Priorities

The occupancy models showed that cattail density plays an important role in determining the occurrence of *F*. *paludicola*. This may explain why so few marshes within the species’ range actually shelter the species. Often, wetlands with high biomass have dense vegetation structure and thick accumulations of leaf-litter, whereas plant diversity is highest where soil nutrients and biomass are low [1], [94]. Rejmankova et al. [95] also found that plant species richness reaches a maximum at intermediate levels of biomass in cattail marshes. Therefore it is reasonable to suggest that high cattail density is associated with low vegetation heterogeneity [96], [97], [98].

Cattail proliferation may be related to anthropic activities as it is a pioneer species in wetland colonization [1]. The removal of riverside forests intensifies soil erosion and the production of sediments that accumulate in the lower parts of the drainage system. This siltation results in the water spreading out, forming environments suitable for the growth of cattail and also exotic plants adapted to saturated soils, such as *Hedychium coronarium* [4]. Cattail also benefits from the eutrophication of aquatic environments and this can result in the cattail dense stands often found near highways, water reservoirs and pastures [98]. However, according to Keddy [1], dense single species stands are often undesirable for wildlife. Therefore, these areas may not be suitable for *F*. *paludicola*. Many of these areas were probably formed comparatively recently and so there may have insufficient time for them to be colonized due to their distance from the generally isolated marshes inhabited by *F*. *paludicola*. Our results show that marshes with higher plant diversity are more suitable for the occurrence of *F*. *paludicola*. Furthermore, we present important information for prioritization of areas in conservation efforts. Our results are also consistent with the idea of maintaining marshes with heterogeneous emergent vegetation, and other sources of plant diversity such as shrub borders, as an important means of wildlife conservation [99].

## Conclusions

The multimodel/field surveys approached helped to indicate the species conservation status and also its environmental requirements in two different scales. We encourage the use of distribution models combined with field validation and occupancy models to assess the threatening status of other species and of their environment. This could be applied worldwide, generating reliable data which could guide successful conservation strategies.

In this study we have shown that besides being restricted, the distribution of *F*. *paludicola* is also very sparse, fragmented and habitat-specific. Based on these distribution patterns and the area of occupancy, *F*. *paludicola* should be considered Critically Endangered according to the IUCN Red List criteria. We also recorded a few local extinctions. We therefore encourage the creation of a network of protected areas in the Upper Tietê and Upper Paraíba do Sul basins, prioritizing the protection of older marshes with heterogeneous vegetation. A series of protected wetlands could be an attraction for tourism and environmental education. The protection of these wetlands would also safeguard water supply and quality, recharge aquifers and protect against flooding. Besides benefiting the human population, the conservation of *F*. *paludicola* could help to restore a piece of Atlantic Forest wetlands’ and maintain its associated biota.

## Supporting Information

S1 Appendix R Script used to perform species distribution models. (DOCX)

S2 Appendix R script used to perform occupancy models. (DOCX)

S1 Dataset Detection history of São Paulo Marsh Antwren in 47 areas (.csv). In column “Detection history”: 1 = detected; 0 = not-detected. In column regarding presence of forest matrix, Eucalyptus plantation and mining: 1 = present; 0 = absent. Water flow was measured by a relative raster value (determined by accumulating the weight for all cells of the raster that flow into each downslope cell).
(CSV)

S1 Table Localities where *Formicivora paludicola* historically occurred (used for distribution modeling procedures) and localities where *F*. *paludicola* currently occurs. *New occurrences.
(DOCX)

S2 Table Procedures used to run each species distribution model. SDM = Species Distribution Model; GLM = generalized linear model; GAM = generalized additive model; MARS = multivariate adaptive regression; CTA = classification tree analysis; ANN = artificial neural networks; RF = random forest.
(DOCX)

S3 Table Localities used for validation process and occupancy models in which *Formicivora paludicola* was not detected. (DOCX)

S4 Table Detection history of São Paulo Marsh Antwren in 47 areas. In column “Detection history”: 1 = detected; 0 = not-detected. In column regarding presence of forest matrix, Eucalyptus plantation and mining: 1 = present; 0 = absent. Water flow was measured by a relative raster value (determined by accumulating the weight for all cells of the raster that flow into each downslope cell).
(DOCX)

S5 Table All occupancy models tested. Density = *Typha dominguensis* density; matrix = presence of forest matrix adjacent to the marsh; area = marsh size; height = *Typha dominguensis* height; minwater = minimum water flow; *Eucalyptus* = presence of *Eucalyptus* plantation adjacent to the marsh; mining = presence of mining activities adjacent to the marsh; rivers = distance to rivers; city = distance to urban areas; highways = distance to highways; maxwater = maximum water flow; avewater = average water flow.
(DOCX)
