# Supplementary material for: A Multiscale Approach Indicates a Severe Reduction in Atlantic Forest Wetlands and Highlights that São Paulo Marsh Antwren Is on the Brink of Extinction
Source: PLoS One. 2015 Mar 23;10(3):e0121315. doi: 10.1371/journal.pone.0121315 (PMC4370614; doi:10.1371/journal.pone.0121315)
Supplement: S1 Appendix — (DOCX) [file pone.0121315.s001.docx]

#############################################################################

#############################################################################

################ #########Species Distribution Modeling ###########################

#########by Glaucia Del-Rio

######Loading Packages

require(biomod2,dismo,foreign,gam,Hmisc,mda,nnet,nlme,plyr,raster,rasterVIS,rgdal,rpart)

######Data of *Formicivora paludicola*

##Presence only

Sp1=rep(1,15)

##Geographic coordinates

x=c(-46.1,-45.97,-46,-46.1,-45.95,-45.92,-45.95,-45.93,-45.8,-46,-46.03,-46.22,-46.15,-46.17,-46.23)

y=c(-23.53,-23.57,-23.57,-23.65,-23.52,-23.52,-23.52,-23.53,-23.57,-23.08,-23.07,-23.4,-23.37,-23.36,-23.38)

DataSpecies=data.frame(Sp1,x,y)

# #the presence/absence data for our species

myResp <- as.numeric(DataSpecies$Sp1)

##the XY coordinates of species data

myRespXY <- DataSpecies[,c("x","y")]

##load the environmental raster layers

myExpl<-stack("alt.asc", "bio3.asc","bio4.asc","bio7.asc","bio14.asc","bio15.asc","dens_dren.asc","hand50.asc")

#############################################################################

######GLM

myRespName<-"GLM"

##The name of the studied species

myBiomodData_GLM <- BIOMOD_FormatingData(resp.var=myResp,expl.var=myExpl,resp.xy=myRespXY,resp.name=myRespName, PA.nb.rep=1,PA.nb.absences=10000,PA.strategy="random")

## set model options

myBiomodOption_GLM <- BIOMOD_ModelingOptions(GLM=list(type="polynomial",interaction.level=0,test="AIC",family="binomial"))

## performing model

myBiomodModelOut_GLM <- BIOMOD_Modeling(myBiomodData_GLM, models="GLM",model.options=myBiomodOption_GLM,NbRunEval=3,DATASplit=70,Prevalence=NULL,VarImport=100,models.eval.meth=c("TSS","ROC","KAPPA"),SaveObj=T,rescal.all.models=T,do.full.models=T,modeling.ide="GLM")

## print model evaluation

(myBiomodModelEval_GLM <- get_evaluations(myBiomodModelOut_GLM))

## projection

myBiomodProj_GLM <- BIOMOD_Projection(

modeling.output = myBiomodModelOut_GLM,

new.env = myExpl,

proj.name = 'GLM',

selected.models = 'all',

binary.meth = NULL,

compress = 'xz',

clamping.mask = F,

output.format = '.img')

#############################################################################

######GAM

myRespName<-"GAM"

## Data

myBiomodData_GAM <- BIOMOD_FormatingData(resp.var=myResp,expl.var=myExpl,resp.xy=myRespXY,resp.name=myRespName, PA.nb.rep=1,PA.nb.absences=10000,PA.strategy="random")

## set model options

myBiomodOption_GAM <- BIOMOD_ModelingOptions(GAM=list(type="s_smoother",interaction.level=0,myFormula=NULL,test="AIC",family="binomial"))

## performing model

myBiomodModelOut_GAM <- BIOMOD_Modeling(myBiomodData_GAM, models=c("GAM"),model.options=myBiomodOption_GAM,NbRunEval=3,DATASplit=70,Prevalence=NULL,VarImport=100,models.eval.meth=c("TSS","ROC","KAPPA"),SaveObj=T,rescal.all.models=T,do.full.models=T,modeling.ide="GAM")

## print model evaluation

(myBiomodModelEval_GAM <- get_evaluations(myBiomodModelOut_GAM))

## projection

myBiomodProj_GAM <- BIOMOD_Projection(

modeling.output = myBiomodModelOut_GAM,

new.env = myExpl,

proj.name = 'GAM',

selected.models = 'all',

binary.meth = 'TSS',

compress = 'xz',

clamping.mask = F,

output.format = '.img')

#############################################################################

######MARS

myRespName<-"MARS"

myBiomodData_MARS <- BIOMOD_FormatingData(resp.var=myResp,expl.var=myExpl,resp.xy=myRespXY,resp.name=myRespName, PA.nb.rep=1,PA.nb.absences=10000,PA.strategy="random")

## set model options

myBiomodOption_MARS <- BIOMOD_ModelingOptions(MARS=list(degree=2,nk=NULL,penalty=2))

## performing model

myBiomodModelOut_MARS <- BIOMOD_Modeling(myBiomodData_MARS, models=c("MARS"),model.options=myBiomodOption_MARS,NbRunEval=3,DATASplit=70,Prevalence=NULL,VarImport=100,models.eval.meth=c("TSS","ROC","KAPPA"),SaveObj=T,rescal.all.models=T,do.full.models=T,modeling.ide="MARS")

## print model evaluation

(myBiomodModelEval_MARS <- get_evaluations(myBiomodModelOut_MARS))

## projection

myBiomodProj_MARS <- BIOMOD_Projection(

modeling.output = myBiomodModelOut_MARS,

new.env = myExpl,

proj.name = 'MARS',

selected.models = 'all',

binary.meth = 'TSS',

compress = 'xz',

clamping.mask = F,

output.format = '.img')

#############################################################################

######CTA

myRespName<-"CTA"

myBiomodData_CTA <- BIOMOD_FormatingData(resp.var=myResp,expl.var=myExpl,resp.xy=myRespXY,resp.name=myRespName, PA.nb.rep=1,PA.nb.absences=15,PA.strategy="disk",PA.dist.min=2)

## setting model options

myBiomodOption_CTA <- BIOMOD_ModelingOptions(CTA=list(method="class",cost=NULL,parms="default"))

## performing model

myBiomodModelOut_CTA <- BIOMOD_Modeling(myBiomodData_CTA, models=c("CTA"),model.options=myBiomodOption_CTA,NbRunEval=3,DATASplit=70,Prevalence=NULL,VarImport=100,models.eval.meth=c("TSS","ROC","KAPPA"),SaveObj=T,rescal.all.models=T,do.full.models=T,modeling.ide="CTA")

## print model evaluation

(myBiomodModelEval_CTA <- get_evaluations(myBiomodModelOut_CTA))

## projection

myBiomodProj_CTA <- BIOMOD_Projection(

modeling.output = myBiomodModelOut_CTA,

new.env = myExpl,

proj.name = 'CTA',

selected.models = 'all',

binary.meth = NULL,

compress = 'xz',

clamping.mask = F,

output.format = '.img')

#############################################################################

######RF

myRespName<-"RF"

myBiomodData_RF <- BIOMOD_FormatingData(resp.var=myResp,expl.var=myExpl,resp.xy=myRespXY,resp.name=myRespName, PA.nb.rep=1,PA.nb.absences=15,PA.strategy="random")

myBiomodOption_RF <- BIOMOD_ModelingOptions(RF=list(ntre=1000,mtry=16))

myBiomodModelOut_RF <- BIOMOD_Modeling(myBiomodData_RF, models=c("RF"),model.options=myBiomodOption_RF,NbRunEval=3,DATASplit=70,Prevalence=NULL,VarImport=100,models.eval.meth=c("TSS","ROC","KAPPA"),SaveObj=T,rescal.all.models=T,do.full.models=T,modeling.ide="RF4")

(myBiomodModelEval_RF <- get_evaluations(myBiomodModelOut_RF))

myBiomodProj_RF <- BIOMOD_Projection(

modeling.output = myBiomodModelOut_RF,

new.env = myExpl,

proj.name = 'RF',

selected.models = 'all',

binary.meth = NULL,

compress = 'xz',

clamping.mask = F,

output.format = '.img')

#############################################################################

######ANN

myRespName<-"ANN"

myBiomodData_ANN <- BIOMOD_FormatingData(resp.var=myResp,expl.var=myExpl,resp.xy=myRespXY,resp.name=myRespName, PA.nb.rep=1,PA.nb.absences=10000,PA.strategy="disk",PA.dist.min=2)

myBiomodOption_ANN <- BIOMOD_ModelingOptions(ANN=list(decay=0.03,size=1,NbCV=7))

myBiomodModelOut_ANN <- BIOMOD_Modeling(myBiomodData_ANN, models=c("ANN"),model.options=myBiomodOption_ANN,NbRunEval=3,DATASplit=70,Prevalence=NULL,VarImport=100,models.eval.meth=c("TSS","ROC","KAPPA"),SaveObj=T,rescal.all.models=T,do.full.models=T,modeling.ide="ANN")

(myBiomodModelEval_ANN <- get_evaluations(myBiomodModelOut_ANN))

myBiomodProj_ANN <- BIOMOD_Projection(

modeling.output = myBiomodModelOut_ANN,

new.env = myExpl,

proj.name = 'ANN',

selected.models = 'all',

binary.meth = 'TSS',

compress = 'xz',

clamping.mask = F,

output.format = '.img')

#############################################################################

#############################################################################

#############################################################################
