# Supplementary material for: A Multiscale Approach Indicates a Severe Reduction in Atlantic Forest Wetlands and Highlights that São Paulo Marsh Antwren Is on the Brink of Extinction
Source: PLoS One. 2015 Mar 23;10(3):e0121315. doi: 10.1371/journal.pone.0121315 (PMC4370614; doi:10.1371/journal.pone.0121315)
Supplement: S2 Appendix — (DOCX) [file pone.0121315.s002.docx]

##########################################################################

##########Occupancy - single species, single season – Formicivora paludicola ##########

###by Glaucia Del-Rio

library(unmarked)

##Data

Data<-read.table(file="S1_Dataset.csv", header=TRUE,sep=";")

head(Data)

## Checking for correlation among between variables:

Datacorrelation=data.frame(Data[,c(7,8,9,10,11,12,13,14,15,16,17,18)])

correlation=cor(Datacorrelation)

m=as.matrix(correlation)

m[lower.tri(m,diag=TRUE)]=NA #Prepare to drop duplicates and meaningless information

m=as.data.frame(as.table(m)) #Turn into a 3-column table

m=na.omit(m) #Get rid of the junk we flagged above

m=m[order(-abs(m$Freq)),] #So

m

write.table(m,"Correlationunmarked.csv",sep=";")

#############################################################################

## Preparing presence/absence data

F.paludicola <- Data[,c("v1", "v2","v3")]

## Normalizing continuous variables to z scores

# Typha dominguensis height

Typha.height.mean<- mean(Data$Typha.dominguensis.height)

Typha.height.sd<-sd(Data$Typha.dominguensis.height)

Typha.height.z<-(Data$Typha.dominguensis.height-Typha.height.mean)/Typha.height.sd

# Typha dominguensis density

Typha.density.mean<- mean(Data$Typha.dominguensis.density)

Typha.density.sd<-sd(Data$Typha.dominguensis.density)

Typha.density.z<-(Data$Typha.dominguensis.density-Typha.density.mean)/Typha.density.sd

# Distance to rivers

Distance.river.mean<- mean(Data$Distance.to.rivers)

Distance.river.sd<-sd(Data$Distance.to.rivers)

Distance.river.z<-(Data$Distance.to.rivers-Distance.river.mean)/Distance.river.sd

# Distance to urban areas

Distance.city.mean = mean(Data$Distance.to.urban.areas)

Distance.city.sd=sd(Data$Distance.to.urban.areas)

Distance.city.z=(Data$Distance.to.urban.areas-Distance.city.mean)/Distance.city.sd

# Distance to highways

Distance.highways.mean<- mean(Data$Distance.to.highways)

Distance.highways.sd<-sd(Data$Distance.to.highways)

Distance.highways.z<-(Data$Distance.to.highways-Distance.highways.mean)/Distance.highways.sd

# Minimum water flow

Min.water.mean <- mean(Data$Minimum.water.flow)

Min.water.sd<-sd(Data$Minimum.water.flow)

Min.water.z<-(Data$Minimum.water.flow-Min.water.mean)/Min.water.sd

# Maximum water flow

Max.water.mean <- mean(Data$Maximum.water.flow)

Max.water.sd<-sd(Data$Maximum.water.flow)

Max.water.z<-(Data$Maximum.water.flow-Max.water.mean)/Max.water.sd

# Average water flow

Ave.water.mean <- mean(Data$Average.water.flow)

Ave.water.sd<-sd(Data$Average.water.flow)

Ave.water.z<-(Data$Average.water.flow-Ave.water.mean)/Ave.water.sd

# Area size

Area.mean<- mean(Data$Area.size)

Area.sd<-sd(Data$Area.size)

Area.z<-(Data$Area.size-Area.mean)/Area.sd

# Creating an unmarked object

Data.umf <- unmarkedFrameOccu(y=F.paludicola, siteCovs=data.frame(matrix=Data$Presence.of.native.forest.matrix, Eucalyptus=Data$Presence.of.Eucalyptus.plantation,mining=Data$Presence.of.mining,height=Typha.height.z,density=Typha.density.z,rivers=Distance.river.z, city=Distance.city.z, highways=Distance.highways.z,minwater=Min.water.z,maxwater=Max.water.z,avewater=Ave.water.z,area=Area.z))

##Nested Models

(M1=occu(~1~density+height+matrix+Eucalyptus+mining+rivers+city+highways+minwater+maxwater+avewater+area,Data.umf))#13 coef

#############################################################

(M2=occu(~1~density+height+matrix+Eucalyptus+mining+rivers+city+highways+minwater+maxwater+avewater,Data.umf)) #12 coef

#############################################################

(M3=occu(~1~density+height+matrix+Eucalyptus+mining+rivers+city+highways+minwater+maxwater,Data.umf)) #11 coef

#############################################################

(M4=occu(~1~density+height+matrix+Eucalyptus+mining+rivers+city+highways+minwater,Data.umf)) #10 coef

#############################################################

(M5=occu(~1~density+height+matrix+Eucalyptus+mining+rivers+city+highways,Data.umf)) #9 coef

#############################################################

(M6=occu(~1~density+height+matrix+Eucalyptus+mining+rivers+city,Data.umf)) #8 coef

#############################################################

(M7=occu(~1~density+height+matrix+Eucalyptus+mining+rivers,Data.umf)) #7 coef

#############################################################

(M8=occu(~1~density+height+matrix+Eucalyptus+mining,Data.umf)) #6 coef

#############################################################

(M9=occu(~1~density+height+matrix+Eucalyptus,Data.umf)) #5 coef

#############################################################

(M10=occu(~1~density+height+matrix,Data.umf))#4 coef

#############################################################

(M11=occu(~1~density+height,Data.umf))#3 coef

#############################################################

(M12=occu(~1~density+matrix,Data.umf))

#############################################################

(M13=occu(~1~density+Eucalyptus,Data.umf))

#############################################################

(M14=occu(~1~density+mining,Data.umf))

#############################################################

(M15=occu(~1~density+rivers,Data.umf))

#############################################################

(M16=occu(~1~density+city,Data.umf))

#############################################################

(M17=occu(~1~density+highways,Data.umf))

#############################################################

(M18=occu(~1~density+minwater,Data.umf))

#############################################################

(M19=occu(~1~density+maxwater,Data.umf))

#############################################################

(M20=occu(~1~density+avewater,Data.umf))

#############################################################

(M21=occu(~1~density+area,Data.umf))

#############################################################

(M22=occu(~1~height+matrix,Data.umf))

#############################################################

(M23=occu(~1~height+Eucalyptus,Data.umf))

#############################################################

(M24=occu(~1~height+mining,Data.umf))

#############################################################

(M25=occu(~1~height+rivers,Data.umf))

#############################################################

(M26=occu(~1~height+city,Data.umf))

#############################################################

(M27=occu(~1~height+highways,Data.umf))

#############################################################

(M28=occu(~1~height+minwater,Data.umf))

#############################################################

(M29=occu(~1~height+maxwater,Data.umf))

#############################################################

(M30=occu(~1~height+avewater,Data.umf))

#############################################################

(M31=occu(~1~height+area,Data.umf))

#############################################################

(M32=occu(~1~matrix+Eucalyptus,Data.umf))

#############################################################

(M33=occu(~1~matrix+mining,Data.umf))

#############################################################

(M34=occu(~1~matrix+rivers,Data.umf))

#############################################################

(M35=occu(~1~matrix+city,Data.umf))

#############################################################

(M36=occu(~1~matrix+highways,Data.umf))

#############################################################

(M37=occu(~1~matrix+minwater,Data.umf))

#############################################################

(M38=occu(~1~matrix+maxwater,Data.umf))

#############################################################

(M39=occu(~1~matrix+avewater,Data.umf))

#############################################################

(M40=occu(~1~matrix+area,Data.umf))

#############################################################

(M41=occu(~1~Eucalyptus+mining,Data.umf))

#############################################################

(M42=occu(~1~Eucalyptus+rivers,Data.umf))

#############################################################

(M43=occu(~1~Eucalyptus+city,Data.umf))

#############################################################

(M44=occu(~1~Eucalyptus+highways,Data.umf))

#############################################################

(M45=occu(~1~Eucalyptus+minwater,Data.umf))

#############################################################

(M46=occu(~1~Eucalyptus+maxwater,Data.umf))

#############################################################

(M47=occu(~1~Eucalyptus+avewater,Data.umf))

#############################################################

(M48=occu(~1~Eucalyptus+area,Data.umf))

#############################################################

(M49=occu(~1~mining+rivers,Data.umf))

#############################################################

(M50=occu(~1~mining+city,Data.umf))

#############################################################

(M51=occu(~1~mining+highways,Data.umf))

#############################################################

(M52=occu(~1~mining+minwater,Data.umf))

#############################################################

(M53=occu(~1~mining+maxwater,Data.umf))

#############################################################

(M54=occu(~1~mining+avewater,Data.umf))

#############################################################

(M55=occu(~1~mining+area,Data.umf))

#############################################################

(M56=occu(~1~rivers+city,Data.umf))

#############################################################

(M57=occu(~1~rivers+highways,Data.umf))

#############################################################

(M58=occu(~1~rivers+minwater,Data.umf))

#############################################################

(M59=occu(~1~rivers+maxwater,Data.umf))

#############################################################

(M60=occu(~1~rivers+avewater,Data.umf))

#############################################################

(M61=occu(~1~rivers+area,Data.umf))

#############################################################

(M62=occu(~1~city+highways,Data.umf))

#############################################################

(M63=occu(~1~city+minwater,Data.umf))

#############################################################

(M64=occu(~1~city+maxwater,Data.umf))

#############################################################

(M65=occu(~1~city+avewater,Data.umf))

#############################################################

(M66=occu(~1~city+area,Data.umf))

#############################################################

(M67=occu(~1~highways+minwater,Data.umf))

#############################################################

(M68=occu(~1~highways+maxwater,Data.umf))

#############################################################

(M69=occu(~1~highways+avewater,Data.umf))

#############################################################

(M70=occu(~1~highways+area,Data.umf))

#############################################################

(M71=occu(~1~minwater+maxwater,Data.umf))

#############################################################

(M72=occu(~1~minwater+avewater,Data.umf))

#############################################################

(M73=occu(~1~minwater+area,Data.umf))

#############################################################

(M74=occu(~1~maxwater+avewater,Data.umf))

#############################################################

(M75=occu(~1~maxwater+area,Data.umf))

#############################################################

(M76=occu(~1~avewater+area,Data.umf))

#############################################################

(M77=occu(~1~density,Data.umf))

#############################################################

(M78=occu(~1~height,Data.umf))

#############################################################

(M79=occu(~1~matrix,Data.umf))

#############################################################

(M80=occu(~1~Eucalyptus,Data.umf))

#############################################################

(M81=occu(~1~mining,Data.umf))

#############################################################

(M82=occu(~1~rivers,Data.umf))

#############################################################

(M83=occu(~1~city,Data.umf))

#############################################################

(M84=occu(~1~highways,Data.umf))

#############################################################

(M85=occu(~1~minwater,Data.umf))

#############################################################

(M86=occu(~1~maxwater,Data.umf))

#############################################################

(M87=occu(~1~avewater,Data.umf))

#############################################################

(M88=occu(~1~area,Data.umf))

#############################################################

(M89=occu(~1~density+height+highways,Data.umf))

#############################################################

(M90=occu(~1~density+height+highways+Eucalyptus,Data.umf))

#############################################################

(M91=occu(~1~density+height+highways+Eucalyptus+maxwater,Data.umf))

#############################################################

(M92=occu(~1~density+height+highways+Eucalyptus+maxwater+city,Data.umf))

###Fitting models

models=fitList("density+height+matrix+Eucalyptus+mining+rivers+city+highways+minwater+maxwater+avewater+area"=M1,"density+height+matrix+Eucalyptus+mining+rivers+city+highways+minwater+maxwater+avewater"=M2,"density+height+matrix+Eucalyptus+mining+rivers+city+highways+minwater+maxwater"=M3,"density+height+matrix+Eucalyptus+mining+rivers+city+highways+minwater)"=M4,"density+height+matrix+Eucalyptus+mining+rivers+city+highways)"=M5,"density+height+matrix+Eucalyptus+mining+rivers+city)"=M6,"density+height+matrix+Eucalyptus+mining+rivers"=M7,"density+height+matrix+Eucalyptus+mining"=M8,"density+height+matrix+Eucalyptus"=M9,"density+height+matrix"=M10,"density+height"=M11,"density+matrix"=M12,"density+Eucalyptus"=M13,"density+mining"=M14,"density+rivers"=M15,"density+city"=M16,"density+highways"=M17,"density+minwater"=M18,"density+maxwater"=M19,"density+avewater"=M20,"density+area"=M21,"height+matrix"=M22,"height+Eucalyptus"=M23,"height+mining"=M24,"height+rivers"=M25,"height+city"=M26,"height+highways"=M27,"height+minwater"=M28,"height+maxwater"=M29,"height+avewater"=M30,"height+area"=M31,"matrix+Eucalyptus"=M32,"matrix+mining"=M33,"matrix+rivers"=M34,"matrix+city"=M35,"matrix+highways"=M36,"matrix+minwater"=M37,"matrix+maxwater"=M38,"matrix+avewater"=M39,"matrix+area"=M40,"Eucalyptus+mining"=M41,"Eucalyptus+rivers"=M42,"Eucalyptus+city"=M43,"Eucalyptus+highways"=M44,"Eucalyptus+minwater"=M45,"Eucalyptus+maxwater"=M46,"Eucalyptus+avewater"=M47,"Eucalyptus+area"=M48,"mining+rivers"=M49,"mining+city"=M50,"mining+highways"=M51,"mining+minwater"=M52,"mining+maxwater"=M53,"mining+avewater"=M54,"mining+area"=M55,"rivers+city"=M56,"rivers+highways"=M57,"rivers+minwater"=M58,"rivers+maxwater"=M59,"rivers+avewater"=M60,"rivers+area"=M61,"city+highways"=M62,"city+minwater"=M63,"city+maxwater"=M64,"city+avewater"=M65,"city+area"=M66,"highways+minwater"=M67,"highways+maxwater"=M68,"highways+avewater"=M69,"highways+area"=M70,"minwater+maxwater"=M71,"minwater+avewater"=M72,"minwater+area"=M73,"maxwater+avewater"=M74,"maxwater+area"=M75,"avewater+area"=M76,"density"=M77,"height"=M78,"matrix"=M79,"Eucalyptus"=M80,"mining"=M81,"rivers"=M82,"city"=M83,"highways"=M84,"minwater"=M85,"maxwater"=M86,"avewater"=M87,"area"=M88,"density+height+highways"=M89,"density+height+highways+Eucalyptus"=M90,"density+height+highways+Eucalyptus+maxwater"=M91,"density+height+highways+Eucalyptus+maxwater+city"=M92)

(list <- modSel(models))

#############################################################################

###Fitting models which converge and have reliable confidence intervals

modelos_which_converge=fitList("density+height"=M11,"density+Eucalyptus"=M13,"density+mining"=M14,"density+rivers"=M15,"density+city"=M16,"density+highways"=M17,"density+maxwater"=M19,"density+avewater"=M20,"density+area"=M21,"height+Eucalyptus"=M23,"height+mining"=M24,"height+rivers"=M25,"height+city"=M26,"height+highways"=M27,"height+maxwater"=M29,"height+avewater"=M30,"height+area"=M31,"Eucalyptus+mining"=M41,"Eucalyptus+rivers"=M42,"Eucalyptus+city"=M43,"Eucalyptus+highways"=M44,"Eucalyptus+maxwater"=M46,"Eucalyptus+avewater"=M47,"Eucalyptus+area"=M48,"mining+rivers"=M49,"mining+city"=M50,"mining+highways"=M51,"mining+maxwater"=M53,"mining+avewater"=M54,"mining+area"=M55,"rivers+city"=M56,"rivers+highways"=M57,"rivers+maxwater"=M59,"rivers+avewater"=M60,"rivers+area"=M61,"city+highways"=M62,"city+maxwater"=M64,"city+avewater"=M65,"city+area"=M66,"highways+maxwater"=M68,"highways+avewater"=M69,"highways+area"=M70,"maxwater+avewater"=M74,"maxwater+area"=M75,"avewater+area"=M76,"density"=M77,"height"=M78,"Eucalyptus"=M80,"mining"=M81,"rivers"=M82,"city"=M83,"highways"=M84,"maxwater"=M86,"avewater"=M87,"area"=M88,"density+height+highways"=M89,"density+height+highways+Eucalyptus"=M90,"density+height+highways+Eucalyptus+maxwater"=M91)

(list2 <- modSel(modelos_which_converge))

#############################################################################

####Model Averaging

require(AICcmodavg)

#############################################################################

####Getting averaged betas

#Typha dominguensis density

modavg(cand.set=list(M77,M11,M17,M13,M19,M16,M14,M15,M21,M89,M20,M90,M91,M64,M29,M86,M75,M53,M46,M68,M59,M74,M30,M65,M31,M87,M76,M78,M26,M69,M54,M47,M60,M24,M66,M88,M23,M25,M27,M55,M61,M48,M70,M83,M81,M82,M80,M84,M50,M62,M43,M56,M41,M51,M49,M42,M57,M44),parm="density",parm.type="psi")

#Typha dominguensis height

modavg(cand.set=list(M77,M11,M17,M13,M19,M16,M14,M15,M21,M89,M20,M90,M91,M64,M29,M86,M75,M53,M46,M68,M59,M74,M30,M65,M31,M87,M76,M78,M26,M69,M54,M47,M60,M24,M66,M88,M23,M25,M27,M55,M61,M48,M70,M83,M81,M82,M80,M84,M50,M62,M43,M56,M41,M51,M49,M42,M57,M44),parm="height",parm.type="psi")

#Distance to highways

modavg(cand.set=list(M77,M11,M17,M13,M19,M16,M14,M15,M21,M89,M20,M90,M91,M64,M29,M86,M75,M53,M46,M68,M59,M74,M30,M65,M31,M87,M76,M78,M26,M69,M54,M47,M60,M24,M66,M88,M23,M25,M27,M55,M61,M48,M70,M83,M81,M82,M80,M84,M50,M62,M43,M56,M41,M51,M49,M42,M57,M44),parm="highways",parm.type="psi")

#Presence of Eucalyptus plantations

modavg(cand.set=list(M77,M11,M17,M13,M19,M16,M14,M15,M21,M89,M20,M90,M91,M64,M29,M86,M75,M53,M46,M68,M59,M74,M30,M65,M31,M87,M76,M78,M26,M69,M54,M47,M60,M24,M66,M88,M23,M25,M27,M55,M61,M48,M70,M83,M81,M82,M80,M84,M50,M62,M43,M56,M41,M51,M49,M42,M57,M44),parm="Eucalyptus",parm.type="psi")

#Maximum water flow

modavg(cand.set=list(M77,M11,M17,M13,M19,M16,M14,M15,M21,M89,M20,M90,M91,M64,M29,M86,M75,M53,M46,M68,M59,M74,M30,M65,M31,M87,M76,M78,M26,M69,M54,M47,M60,M24,M66,M88,M23,M25,M27,M55,M61,M48,M70,M83,M81,M82,M80,M84,M50,M62,M43,M56,M41,M51,M49,M42,M57,M44),parm="maxwater",parm.type="psi")

#Distance to urban areas

modavg(cand.set=list(M77,M11,M17,M13,M19,M16,M14,M15,M21,M89,M20,M90,M91,M64,M29,M86,M75,M53,M46,M68,M59,M74,M30,M65,M31,M87,M76,M78,M26,M69,M54,M47,M60,M24,M66,M88,M23,M25,M27,M55,M61,M48,M70,M83,M81,M82,M80,M84,M50,M62,M43,M56,M41,M51,M49,M42,M57,M44),parm="city",parm.type="psi")

#Presence of mining

modavg(cand.set=list(M77,M11,M17,M13,M19,M16,M14,M15,M21,M89,M20,M90,M91,M64,M29,M86,M75,M53,M46,M68,M59,M74,M30,M65,M31,M87,M76,M78,M26,M69,M54,M47,M60,M24,M66,M88,M23,M25,M27,M55,M61,M48,M70,M83,M81,M82,M80,M84,M50,M62,M43,M56,M41,M51,M49,M42,M57,M44),parm="mining",parm.type="psi")

#Distance to rivers

modavg(cand.set=list(M77,M11,M17,M13,M19,M16,M14,M15,M21,M89,M20,M90,M91,M64,M29,M86,M75,M53,M46,M68,M59,M74,M30,M65,M31,M87,M76,M78,M26,M69,M54,M47,M60,M24,M66,M88,M23,M25,M27,M55,M61,M48,M70,M83,M81,M82,M80,M84,M50,M62,M43,M56,M41,M51,M49,M42,M57,M44),parm="rivers",parm.type="psi")

#Marshes size

modavg(cand.set=list(M77,M11,M17,M13,M19,M16,M14,M15,M21,M89,M20,M90,M91,M64,M29,M86,M75,M53,M46,M68,M59,M74,M30,M65,M31,M87,M76,M78,M26,M69,M54,M47,M60,M24,M66,M88,M23,M25,M27,M55,M61,M48,M70,M83,M81,M82,M80,M84,M50,M62,M43,M56,M41,M51,M49,M42,M57,M44),parm="area",parm.type="psi")

#Average water flow

modavg(cand.set=list(M77,M11,M17,M13,M19,M16,M14,M15,M21,M89,M20,M90,M91,M64,M29,M86,M75,M53,M46,M68,M59,M74,M30,M65,M31,M87,M76,M78,M26,M69,M54,M47,M60,M24,M66,M88,M23,M25,M27,M55,M61,M48,M70,M83,M81,M82,M80,M84,M50,M62,M43,M56,M41,M51,M49,M42,M57,M44),parm="avewater",parm.type="psi")

#############################################################################

#####Getting averaged psi

fmList <- fitList(M77,M11,M17,M13,M19,M16,M14,M15,M21,M89,M20,M90,M91,M64,M29,M86,M75,M53,M46,M68,M59,M74,M30,M65,M31,M87,M76,M78,M26,M69,M54,M47,M60,M24,M66,M88,M23,M25,M27,M55,M61,M48,M70,M83,M81,M82,M80,M84,M50,M62,M43,M56,M41,M51,M49,M42,M57,M44)

(psi_estimates=predict(fmList, type="state"))

mean(psi_estimates$Predicted)

mean(psi_estimates$SE)

mean(psi_estimates$lower)

mean(psi_estimates$upper)
