# Supplementary material for: A Multiscale Approach Indicates a Severe Reduction in Atlantic Forest Wetlands and Highlights that São Paulo Marsh Antwren Is on the Brink of Extinction
Source: PLoS One. 2015 Mar 23;10(3):e0121315. doi: 10.1371/journal.pone.0121315 (PMC4370614; doi:10.1371/journal.pone.0121315)
Supplement: S1 Table — *New occurrences. (DOCX) [file pone.0121315.s004.docx]

| **Municipality** | **Locality** | **Latitude (S)** | | **Longitude (W)** | | **Used for Modeling** | **Currently Occupied** |
| --- | --- | --- | --- | --- | --- | --- | --- |
| Biritiba-Mirim | Barragem de Ponte Nova | 23 | 23 | 46 | 13 | Yes | No |
| Biritiba-Mirim | Estrada da Casa Grande | 23 | 34 | 46 | 0 | Yes | Yes |
| Mogi das Cruzes | Avenida Presidente Castelo branco com Estrada do Rio Acima | 23 | 32 | 46 | 6 | Yes | Yes |
| Mogi das Cruzes | Barragem do Rio Biritiba 1 | 23 | 39 | 46 | 6 | Yes | No |
| Mogi das Cruzes | Córrego Taboão do Parateí | 23 | 24 | 46 | 13 | Yes | Yes |
| Mogi das Cruzes | Jardim Guanabara | 23 | 22 | 46 | 9 | Yes | Yes |
| Mogi das Cruzes | Jardim Itapeti | 23 | 21 | 46 | 10 | Yes | Yes |
| Mogi das Cruzes | Fazenda Taboão | 23 | 23 | 46 | 14 | Yes | Yes |
| Salesópolis | Barragem do Paraitinga 1 | 23 | 32 | 45 | 56 | Yes | No |
| Salesópolis | Distrito Nossa Senhora dos Remédios | 23 | 31 | 45 | 57 | Yes | Yes |
| Salesópolis | Rio Paraitinga | 23 | 31 | 45 | 55 | Yes | No |
| Salesópolis | Usina da Light Estrada dos Mirandas (Ribeirão do Pote) | 23 | 34 | 45 | 49 | Yes | Yes |
| Salesópolis* | Parque Usina da Light | 23 | 33 | 45 | 50 | No | Yes |
| São José dos Campos | Fazenda Montes Claros | 23 | 4 | 46 | 2 | Yes | Yes |
| São José dos Campos | Sítio Caleb | 23 | 5 | 46 | 0 | Yes | Yes |
| Guararema* | Fazenda Suzano 1 | 23 | 25 | 45 | 58 | No | Yes |
| Guararema* | Fazenda Suzano 2 | 23 | 26 | 45 | 59 | No | Yes |
