# Supplementary material for: A Multiscale Approach Indicates a Severe Reduction in Atlantic Forest Wetlands and Highlights that São Paulo Marsh Antwren Is on the Brink of Extinction
Source: PLoS One. 2015 Mar 23;10(3):e0121315. doi: 10.1371/journal.pone.0121315 (PMC4370614; doi:10.1371/journal.pone.0121315)
Supplement: S2 Table — SDM = Species Distribution Model; GLM = generalized linear model; GAM = generalized additive model; MARS = multivariate adaptive regression; CTA = classification tree analysis; ANN = artificial neural networks; RF = random forest. (DOCX) [file pone.0121315.s005.docx]

| **SDM** | **GLM** | **GAM** | **MARS** | **CTA** | **ANN** | **RF** |
| --- | --- | --- | --- | --- | --- | --- |
| **Reference** | Thuiller 2003 [38] | Thuiller 2003 [38] | Muñoz and Felicísimo 2004 [51] | Thuiller 2003 [38] | Thuiller 2003 [38] | Breiman and Cutler 2003 [52] |
| **Options** | • Polynomial terms; • Interactions only for the linear terms; • AIC criterion to choose the best model. | • Smooth splines; • Smoothness selected by cross-validation; • AIC to choose the most parsimonious model. | • Original variables and second order interactions. | • 10-fold cross-validation. | • Seven hidden units in a single hidden layer; • Decay equal to 0.03; • Average of 10 runs used for projections and predictions. | • Number of predictors to be chosen at each tree node = one-third the number of predictors. |
