# Supplementary material for: A Multiscale Approach Indicates a Severe Reduction in Atlantic Forest Wetlands and Highlights that São Paulo Marsh Antwren Is on the Brink of Extinction
Source: PLoS One. 2015 Mar 23;10(3):e0121315. doi: 10.1371/journal.pone.0121315 (PMC4370614; doi:10.1371/journal.pone.0121315)
Supplement: S3 Table — (DOCX) [file pone.0121315.s006.docx]

| **Name** | **longitude** | **latitude** | **Type** |
| --- | --- | --- | --- |
| 5 | -46.80 | -23.90 | Validation (no occurrence) |
| 6 | -46.79 | -23.93 | Validation (no occurrence) |
| 7 | -45.91 | -23.42 | Occupancy Model (no occurrence) |
| 9 | -47.46 | -23.81 | Validation (no occurrence) |
| 10 | -47.22 | -23.65 | Validation (no occurrence) |
| 11 | -47.17 | -23.65 | Validation (no occurrence) |
| 15 | -46.96 | -23.56 | Validation (no occurrence) |
| 16 | -46.62 | -23.10 | Validation (no occurrence) |
| 17 | -46.46 | -23.15 | Validation (no occurrence) |
| 18 | -46.40 | -23.16 | Validation (no occurrence) |
| 19 | -45.90 | -23.19 | Occupancy Model (no occurrence) |
| 20 | -45.93 | -23.23 | Occupancy Model (no occurrence) |
| 21 | -45.97 | -23.23 | Occupancy Model (no occurrence) |
| 24 | -46.65 | -24.09 | Validation (no occurrence) |
| 27 | -46.21 | -23.62 | Occupancy Model (no occurrence) |
| 28 | -46.09 | -23.69 | Occupancy Model (no occurrence) |
| 33 | -46.60 | -23.40 | Validation (no occurrence) |
| 36 | -46.85 | -23.20 | Validation (no occurrence) |
| 38 | -46.72 | -23.15 | Validation (no occurrence) |
| 43 | -45.62 | -23.55 | Occupancy Model (no occurrence) |
| 44 | -45.63 | -23.55 | Occupancy Model (no occurrence) |
| 54 | -46.28 | -23.56 | Occupancy Model (no occurrence) |
| 56 | -47.43 | -23.82 | Validation (no occurrence) |
| 57 | -46.38 | -23.61 | Occupancy Model (no occurrence) |
| 58 | -45.23 | -23.38 | Validation (no occurrence) |
| 60 | -45.21 | -23.37 | Validation (no occurrence) |
| 62 | -45.42 | -22.98 | Validation (no occurrence) |
| 66 | -45.63 | -23.12 | Occupancy Model (no occurrence) |
| 67 | -45.67 | -23.11 | Occupancy Model (no occurrence) |
| 69 | -46.23 | -23.28 | Occupancy Model (no occurrence) |
| 70 | -46.02 | -23.46 | Occupancy Model (no occurrence) |
| 71 | -46.00 | -23.45 | Occupancy Model (no occurrence) |
| 72 | -46.00 | -23.48 | Occupancy Model (no occurrence) |
| 73 | -46.04 | -23.42 | Occupancy Model (no occurrence) |
| 74 | -46.00 | -23.36 | Validation (no occurrence) |
| 76 | -45.93 | -23.42 | Occupancy Model (no occurrence) |
| 79 | -46.05 | -23.49 | Occupancy Model (no occurrence) |
| 81 | -45.84 | -23.48 | Occupancy Model (no occurrence) |
| 82 | -46.12 | -23.37 | Occupancy Model (no occurrence) |
| 83 | -46.08 | -23.35 | Occupancy Model (no occurrence) |
| 84 | -46.01 | -23.49 | Occupancy Model (no occurrence) |
| 85 | -45.99 | -23.48 | Occupancy Model (no occurrence) |
| 86 | -45.98 | -23.44 | Occupancy Model (no occurrence) |
| 103 | -45.86 | -23.40 | Occupancy Model (no occurrence) |
| 104 | -45.28 | -23.32 | Validation (no occurrence) |
| 105 | -46.18 | -23.51 | Occupancy Model (no occurrence) |
| 106 | -46.32 | -23.50 | Occupancy Model (no occurrence) |
| 107 | -46.22 | -23.58 | Occupancy Model (no occurrence) |
| 108 | -46.26 | -23.57 | Occupancy Model (no occurrence) |
| 109 | -46.26 | -23.61 | Occupancy Model (no occurrence) |
| 113 | -45.82 | -22.92 | Validation (no occurrence) |
| 114 | -45.67 | -23.36 | Validation (no occurrence) |
| 115 | -45.71 | -23.26 | Occupancy Model (no occurrence) |
| 116 | -45.79 | -23.29 | Occupancy Model (no occurrence) |
| 117 | -45.72 | -23.37 | Occupancy Model (no occurrence) |
| 118 | -46.17 | -23.43 | Occupancy Model (no occurrence) |
| 119 | -46.37 | -23.47 | Validation (no occurrence) |
| 120 | -46.73 | -23.78 | Validation (no occurrence) |
| 121 | -44.39 | -22.44 | Validation (no occurrence) |
| 122 | -46.83 | -23.66 | Validation (no occurrence) |
| 123 | -44.70 | -22.51 | Validation (no occurrence) |
| 124 | -44.50 | -22.39 | Validation (no occurrence) |
| 125 | -44.44 | -22.44 | Validation (no occurrence) |
| 126 | -44.76 | -22.56 | Validation (no occurrence) |
