# Supplementary material for: A Multiscale Approach Indicates a Severe Reduction in Atlantic Forest Wetlands and Highlights that São Paulo Marsh Antwren Is on the Brink of Extinction
Source: PLoS One. 2015 Mar 23;10(3):e0121315. doi: 10.1371/journal.pone.0121315 (PMC4370614; doi:10.1371/journal.pone.0121315)
Supplement: S4 Table — In column “Detection history”: 1 = detected; 0 = not-detected. In column regarding presence of forest matrix, Eucalyptus plantation and mining: 1 = present; 0 = absent. Water flow was measured by a relative raster value (determined by accumulating the weight for all cells of the raster that flow into each downslope cell). (DOCX) [file pone.0121315.s007.docx]

| **Site ID** | **Coordinates** | | **Detection History** | | | **Typha dominguensis height (mm)** | **Typha dominguensis density** | **Presence of native forest matrix** | **Presence of Eucalyptus plantation** | **Presence of mining** | **Distance to rivers (m)** | **Distance to urban areas (m)** | **Distance to highways (m)** | **Minimum water flow** | **Maximum water flow** | **Average water flow** | **Area (m^2^)** |
| --- | --- | --- | --- | --- | --- | --- | --- | --- | --- | --- | --- | --- | --- | --- | --- | --- | --- |
| 7 | -45.91 | -23.42 | 0 | 0 | 0 | 2922.50 | 23 | 0 | 0 | 0 | 0.91 | 2197.43 | 107.99 | 16.00 | 6313.00 | 3772.80 | 3540.06 |
| 19 | -45.9 | -23.19 | 0 | 0 | 0 | 2217.50 | 36 | 0 | 0 | 0 | 9.54 | 0.00 | 12125.60 | 0.00 | 314.00 | 50.02 | 39282.36 |
| 20 | -45.93 | -23.23 | 0 | 0 | 0 | 2651.25 | 37 | 0 | 0 | 0 | 38.88 | 0.00 | 10771.04 | 0.00 | 212.00 | 25.24 | 82367.07 |
| 21 | -45.95 | -23.22 | 0 | 0 | 0 | 2613.75 | 31 | 0 | 0 | 1 | 65.02 | 3839.07 | 11936.94 | 0.00 | 118.00 | 19.67 | 10120.18 |
| 27 | -46.21 | -23.62 | 0 | 0 | 0 | 2273.50 | 32 | 0 | 0 | 1 | 137.86 | 5000.65 | 69.09 | 0.00 | 62.00 | 9.20 | 33313.50 |
| 28 | -46.09 | -23.69 | 0 | 0 | 0 | 3215.50 | 38 | 1 | 0 | 1 | 12.42 | 9478.83 | 2103.30 | 0.00 | 8937.00 | 918.82 | 34250.44 |
| 30 | -45.97 | -23.43 | 1 | 1 | 1 | 2720.00 | 3 | 1 | 1 | 0 | 17.19 | 5134.68 | 853.99 | 0.00 | 93983.00 | 11895.99 | 220688.10 |
| 41 | -45.84 | -23.56 | 1 | 1 | 1 | 3332.50 | 6 | 1 | 1 | 0 | 9.26 | 2783.09 | 2434.16 | 0.00 | 92873.00 | 11983.04 | 74421.23 |
| 43 | -45.62 | -23.55 | 0 | 0 | 0 | 2596.25 | 31 | 0 | 1 | 0 | 8.41 | 17110.62 | 40.44 | 1012.00 | 1015.00 | 1013.67 | 2909.75 |
| 44 | -45.63 | -23.55 | 0 | 0 | 0 | 2473.75 | 34 | 0 | 1 | 0 | 5.04 | 17001.29 | 38.78 | 42.00 | 1454.00 | 513.00 | 3048.90 |
| 54 | -46.28 | -23.56 | 0 | 0 | 0 | 2555.00 | 35 | 0 | 0 | 1 | 77.64 | 872.34 | 964.61 | 0.00 | 173091.00 | 3077.36 | 1030789.17 |
| 55 | -45.99 | -23.44 | 1 | 1 | 1 | 2672.50 | 8 | 1 | 1 | 1 | 50.95 | 3755.86 | 74.58 | 0.00 | 3283.00 | 279.58 | 43592.51 |
| 57 | -46.38 | -23.61 | 0 | 0 | 0 | 2905.00 | 29 | 1 | 0 | 1 | 168.68 | 9778.00 | 236.92 | 0.00 | 51.00 | 12.20 | 36920.87 |
| 66 | -45.63 | -23.12 | 0 | 0 | 0 | 2818.75 | 29 | 0 | 1 | 0 | 111.17 | 1023.49 | 223.01 | 0.00 | 12893.00 | 810.77 | 43370.61 |
| 67 | -45.67 | -23.11 | 0 | 0 | 0 | 2552.50 | 31 | 0 | 0 | 0 | 1.66 | 1032.12 | 79.54 | 0.00 | 3204.00 | 1599.38 | 6899.03 |
| 69 | -46.23 | -23.28 | 0 | 0 | 0 | 2726.25 | 32 | 0 | 1 | 0 | 11.98 | 1337.02 | 915.53 | 3.00 | 104852.00 | 30653.00 | 21060.08 |
| 70 | -46.02 | -23.46 | 0 | 0 | 0 | 2700.00 | 33 | 0 | 1 | 0 | 14.42 | 3494.71 | 2905.25 | 0.00 | 7334.00 | 2722.13 | 21558.55 |
| 71 | -46 | -23.45 | 0 | 0 | 0 | 2190.00 | 36 | 0 | 0 | 0 | 1.50 | 3837.32 | 125.96 | 0.00 | 2791.00 | 920.67 | 11647.02 |
| 72 | -46 | -23.48 | 0 | 0 | 0 | 2145.00 | 37 | 0 | 1 | 0 | 44.17 | 7068.12 | 19.76 | 0.00 | 29.00 | 12.17 | 4371.36 |
| 73 | -46.04 | -23.42 | 0 | 0 | 0 | 2566.75 | 32 | 0 | 0 | 0 | 56.08 | 466.28 | 131.56 | 0.00 | 244.00 | 24.93 | 12712.75 |
| 76 | -45.93 | -23.42 | 0 | 0 | 0 | 2808.00 | 29 | 1 | 1 | 0 | 62.37 | 4540.09 | 884.80 | 1.00 | 12.00 | 5.67 | 2437.46 |
| 79 | -46.05 | -23.49 | 0 | 0 | 0 | 2800.00 | 31 | 0 | 1 | 0 | 21.39 | 5681.27 | 80.15 | 0.00 | 2674.00 | 251.77 | 11255.47 |
| 81 | -45.84 | -23.48 | 0 | 0 | 0 | 2584.25 | 38 | 0 | 1 | 0 | 55.58 | 4562.36 | 3028.38 | 1.00 | 47.00 | 30.78 | 7736.21 |
| 82 | -46.12 | -23.37 | 0 | 0 | 0 | 2239.00 | 35 | 0 | 1 | 1 | 80.73 | 3722.07 | 1137.75 | 3.00 | 73.00 | 12.06 | 14827.66 |
| 83 | -46.08 | -23.35 | 0 | 0 | 0 | 1896.25 | 36 | 0 | 1 | 0 | 23.82 | 5503.47 | 922.31 | 0.00 | 2432.00 | 396.98 | 35801.54 |
| 84 | -46.01 | -23.49 | 0 | 0 | 0 | 2929.00 | 37 | 0 | 0 | 0 | 10.97 | 7629.37 | 330.10 | 0.00 | 6792.00 | 1182.54 | 41688.05 |
| 85 | -45.99 | -23.48 | 0 | 0 | 0 | 2136.00 | 35 | 0 | 1 | 0 | 12.46 | 7206.61 | 372.25 | 0.00 | 7656.00 | 1375.44 | 65435.81 |
| 86 | -45.98 | -23.44 | 0 | 0 | 0 | 2278.00 | 34 | 0 | 1 | 0 | 30.39 | 4510.73 | 175.00 | 0.00 | 3343.00 | 932.93 | 13900.35 |
| 87 | -45.82 | -23.57 | 1 | 1 | 1 | 2345.00 | 9 | 1 | 1 | 0 | 119.66 | 4543.62 | 4106.06 | 0.00 | 54225.00 | 4455.71 | 290161.29 |
| 88 | -46.12 | -23.54 | 1 | 1 | 1 | 2789.00 | 10 | 1 | 0 | 0 | 174.10 | 3428.77 | 1088.01 | 0.00 | 732674.00 | 20640.31 | 1180524.08 |
| 89 | -46.05 | -23.07 | 1 | 1 | 1 | 2834.00 | 7 | 1 | 1 | 0 | 41.83 | 16701.47 | 2854.67 | 0.00 | 2902.00 | 241.23 | 117738.93 |
| 90 | -45.96 | -23.53 | 1 | 1 | 1 | 2829.00 | 9 | 1 | 0 | 0 | 43.43 | 8802.01 | 2309.79 | 0.00 | 771.00 | 69.90 | 86962.01 |
| 91 | -46.01 | -23.1 | 1 | 1 | 1 | 2100.00 | 8 | 1 | 0 | 0 | 52.52 | 12321.77 | 301.63 | 0.00 | 55245.00 | 6552.19 | 52914.89 |
| 100 | -46.23 | -23.41 | 1 | 1 | 1 | 2931.00 | 6 | 1 | 1 | 0 | 32.87 | 5938.25 | 1696.54 | 0.00 | 32466.00 | 1986.06 | 54096.14 |
| 101 | -46.16 | -23.37 | 1 | 1 | 1 | 2910.00 | 7 | 1 | 0 | 1 | 15.72 | 855.88 | 1317.24 | 0.00 | 26077.00 | 3764.73 | 144171.08 |
| 102 | -46.17 | -23.36 | 1 | 1 | 1 | 3011.00 | 6 | 1 | 0 | 0 | 24.37 | 792.59 | 399.73 | 0.00 | 209677.00 | 22778.05 | 152221.69 |
| 103 | -45.86 | -23.4 | 0 | 0 | 0 | 3332.50 | 31 | 0 | 1 | 0 | 8.83 | 1581.41 | 103.82 | 0.00 | 1319.00 | 338.82 | 9597.09 |
| 105 | -46.18 | -23.51 | 0 | 0 | 0 | 2590.00 | 32 | 0 | 0 | 1 | 16.27 | 262.35 | 60.18 | 0.00 | 40720.00 | 4235.19 | 127140.59 |
| 106 | -46.32 | -23.5 | 0 | 0 | 0 | 2299.75 | 33 | 0 | 0 | 1 | 103.74 | 306.60 | 165.19 | 56.00 | 185.00 | 76.97 | 86649.45 |
| 107 | -46.22 | -23.58 | 0 | 0 | 0 | 2551.50 | 34 | 0 | 0 | 0 | 563.77 | 135.74 | 96.51 | 0.00 | 35.00 | 13.75 | 18470.20 |
| 108 | -46.26 | -23.57 | 0 | 0 | 0 | 2542.50 | 29 | 0 | 0 | 1 | 94.14 | 1485.97 | 81.71 | 0.00 | 42.00 | 3.69 | 12527.81 |
| 109 | -46.26 | -23.61 | 0 | 0 | 0 | 2602.00 | 34 | 0 | 0 | 1 | 51.73 | 242.12 | 3039.78 | 0.00 | 355.00 | 39.40 | 8793.86 |
| 112 | -46.24 | -23.39 | 1 | 1 | 1 | 3013.00 | 32 | 1 | 0 | 0 | 61.80 | 5556.56 | 3910.06 | 0.00 | 14026.00 | 1278.82 | 18481.53 |
| 115 | -45.71 | -23.26 | 0 | 0 | 0 | 2068.75 | 31 | 1 | 1 | 0 | 71.67 | 654.83 | 131.80 | 0.00 | 18.00 | 5.71 | 6748.88 |
| 116 | -45.79 | -23.29 | 0 | 0 | 0 | 2311.00 | 29 | 0 | 1 | 0 | 2.22 | 4724.52 | 118.51 | 0.00 | 4234.00 | 1920.45 | 9149.81 |
| 117 | -45.72 | -23.37 | 0 | 0 | 0 | 2137.50 | 27 | 0 | 1 | 0 | 6.72 | 4856.56 | 2889.10 | 0.00 | 975.00 | 364.60 | 4921.65 |
| 118 | -46.16 | -23.43 | 0 | 0 | 0 | 2867.50 | 25 | 1 | 0 | 0 | 19.34 | 5487.50 | 3523.76 | 0.00 | 5502.00 | 1224.22 | 7269.07 |
