# Supplementary material for: A Multiscale Approach Indicates a Severe Reduction in Atlantic Forest Wetlands and Highlights that São Paulo Marsh Antwren Is on the Brink of Extinction
Source: PLoS One. 2015 Mar 23;10(3):e0121315. doi: 10.1371/journal.pone.0121315 (PMC4370614; doi:10.1371/journal.pone.0121315)
Supplement: S5 Table — Density = Typha dominguensis density; matrix = presence of forest matrix adjacent to the marsh; area = marsh size; height = Typha dominguensis height; minwater = minimum water flow; Eucalyptus = presence of Eucalyptus plantation adjacent to the marsh; mining = presence of mining activities adjacent to the marsh; rivers = distance to rivers; city = distance to urban areas; highways = distance to highways; maxwater = maximum water flow; avewater = average water flow. (DOCX) [file pone.0121315.s008.docx]

| **Models** | **nPars** | **AIC** | **delta** | **AICwt** | **cumltvWt** | **Used models: blue; excluded models: red** |
| --- | --- | --- | --- | --- | --- | --- |
| Ψ(density+matrix)p(.) | 4 | 15.15 | 0 | 1.70E-01 | 0.17 | uninformative confidence intervals |
| Ψ (matrix+area)p(.) | 4 | 16.24 | 1.09 | 9.70E-02 | 0.26 | do not converge |
| Ψ (density+height+matrix)p(.) | 5 | 16.49 | 1.33 | 8.50E-02 | 0.35 | uninformative confidence intervals |
| Ψ (density)p(.) | 3 | 16.7 | 1.55 | 7.70E-02 | 0.43 | M77 |
| Ψ (density+minwater)p(.) | 4 | 17.65 | 2.5 | 4.80E-02 | 0.47 | uninformative confidence intervals |
| Ψ (density+height)p(.) | 4 | 17.68 | 2.53 | 4.70E-02 | 0.52 | M11 |
| Ψ (density+height+matrix+*Eucalyptus*+mining+rivers+city)p(.) | 9 | 18.01 | 2.86 | 4.00E-02 | 0.56 | do not converge |
| Ψ (density+highways)p(.) | 4 | 18.06 | 2.91 | 3.90E-02 | 0.6 | M17 |
| Ψ (density+*Eucalyptus*)p(.) | 4 | 18.24 | 3.09 | 3.60E-02 | 0.63 | M13 |
| Ψ (density+height+matrix+*Eucalyptus*)p(.) | 6 | 18.39 | 3.24 | 3.30E-02 | 0.67 | uninformative confidence intervals |
| Ψ (density+maxwater)p(.) | 4 | 18.46 | 3.31 | 3.20E-02 | 0.7 | M19 |
| Ψ (density+city)p(.) | 4 | 18.49 | 3.33 | 3.10E-02 | 0.73 | M16 |
| Ψ (density+mining)p(.) | 4 | 18.51 | 3.36 | 3.10E-02 | 0.76 | M14 |
| Ψ (density+rivers)p(.) | 4 | 18.61 | 3.45 | 3.00E-02 | 0.79 | M15 |
| Ψ (density+area)p(.) | 4 | 18.63 | 3.47 | 2.90E-02 | 0.82 | M21 |
| Ψ (density+height+highways)p(.) | 5 | 18.67 | 3.52 | 2.90E-02 | 0.85 | M89 |
| Ψ (density+avewater)p(.) | 4 | 18.7 | 3.54 | 2.80E-02 | 0.88 | M20 |
| Ψ (density+height+matrix+*Eucalyptus*+mining+rivers)p(.) | 8 | 18.8 | 3.64 | 2.70E-02 | 0.9 | do not converge |
| Ψ (density+height+matrix+*Eucalyptus*+mining)p(.) | 7 | 19.71 | 4.55 | 1.70E-02 | 0.92 | uninformative confidence intervals |
| Ψ (matrix+maxwater)p(.) | 4 | 19.93 | 4.78 | 1.50E-02 | 0.94 | do not converge |
| Ψ (density+height+matrix+*Eucalyptus*+mining+rivers+city+highways)p(.) | 10 | 20 | 4.85 | 1.50E-02 | 0.95 | uninformative confidence intervals |
| Ψ (density+height+highways+*Eucalyptus*)p(.) | 6 | 20.51 | 5.35 | 1.10E-02 | 0.96 | M90 |
| Ψ (density+height+highways+*Eucalyptus*+maxwater+city)p(.) | 8 | 20.52 | 5.37 | 1.10E-02 | 0.97 | do not converge |
| Ψ (matrix+avewater)p(.) | 4 | 21.37 | 6.21 | 7.50E-03 | 0.98 | uninformative confidence intervals |
| Ψ (density+height+highways+*Eucalyptus*+maxwater)p(.) | 7 | 21.5 | 6.35 | 7.00E-03 | 0.99 | M91 |
| Ψ (density+height+matrix+*Eucalyptus*+mining+rivers+city+highways+minwater)p(.) | 11 | 22 | 6.85 | 5.40E-03 | 0.99 | do not converge |
| Ψ (density+height+matrix+*Eucalyptus*+mining+rivers+city+highways+minwater+maxwater)p(.) | 12 | 24 | 8.85 | 2.00E-03 | 1 | do not converge |
| Ψ (matrix+minwater)p(.) | 4 | 26.06 | 10.91 | 7.10E-04 | 1 | uninformative confidence intervals |
| Ψ (density+height+matrix+*Eucalyptus*+mining+rivers+city+highways+minwater+maxwater+avewater)p(.) | 13 | 26.1 | 10.95 | 7.00E-04 | 1 | do not converge |
| Ψ (matrix)p(.) | 3 | 26.6 | 11.44 | 5.40E-04 | 1 | uninformative confidence intervals |
| Ψ (matrix+mining)p(.) | 4 | 27.59 | 12.44 | 3.30E-04 | 1 | uninformative confidence intervals |
| Ψ (density+height+matrix+*Eucalyptus*+mining+rivers+city+highways+minwater+maxwater+avewater+area)p(.) | 14 | 28 | 12.85 | 2.70E-04 | 1 | do not converge |
| Ψ (matrix+highways)p(.) | 4 | 28.25 | 13.1 | 2.40E-04 | 1 | uninformative confidence intervals |
| Ψ (matrix+rivers)p(.) | 4 | 28.35 | 13.2 | 2.30E-04 | 1 | uninformative confidence intervals |
| Ψ (matrix+*Eucalyptus*)p(.) | 4 | 28.46 | 13.3 | 2.20E-04 | 1 | uninformative confidence intervals |
| Ψ (height+matrix)p(.) | 4 | 28.59 | 13.44 | 2.00E-04 | 1 | uninformative confidence intervals |
| Ψ (matrix+city)p(.) | 4 | 28.6 | 13.44 | 2.00E-04 | 1 | uninformative confidence intervals |
| Ψ (minwater+avewater)p(.) | 4 | 42.4 | 27.25 | 2.00E-07 | 1 | do not converge |
| Ψ (minwater+maxwater)p(.) | 4 | 47.67 | 32.51 | 1.40E-08 | 1 | do not converge |
| Ψ (city+maxwater)p(.) | 4 | 48.6 | 33.45 | 9.10E-09 | 1 | M64 |
| Ψ (height+maxwater)p(.) | 4 | 49.39 | 34.24 | 6.10E-09 | 1 | M29 |
| Ψ (maxwater)p(.) | 3 | 50.22 | 35.07 | 4.00E-09 | 1 | M86 |
| Ψ (maxwater+area)p(.) | 4 | 51.34 | 36.19 | 2.30E-09 | 1 | M75 |
| Ψ (mining+maxwater)p(.) | 4 | 51.62 | 36.46 | 2.00E-09 | 1 | M53 |
| Ψ (*Eucalyptus*+maxwater)p(.) | 4 | 51.95 | 36.8 | 1.70E-09 | 1 | M46 |
| Ψ (highways+maxwater)p(.) | 4 | 52.04 | 36.88 | 1.60E-09 | 1 | M68 |
| Ψ (rivers+maxwater)p(.) | 4 | 52.12 | 36.97 | 1.60E-09 | 1 | M59 |
| Ψ (maxwater+avewater)p(.) | 4 | 52.22 | 37.07 | 1.50E-09 | 1 | M74 |
| Ψ (height+minwater)p(.) | 4 | 52.25 | 37.1 | 1.50E-09 | 1 | do not converge |
| Ψ (height+avewater)p(.) | 4 | 52.54 | 37.39 | 1.30E-09 | 1 | M30 |
| Ψ (city+avewater)p(.) | 4 | 52.65 | 37.5 | 1.20E-09 | 1 | M65 |
| Ψ (height+area)p(.) | 4 | 53.08 | 37.92 | 9.70E-10 | 1 | M31 |
| Ψ (city+minwater)p(.) | 4 | 53.16 | 38.01 | 9.30E-10 | 1 | do not converge |
| Ψ (avewater)p(.) | 3 | 53.45 | 38.29 | 8.10E-10 | 1 | M87 |
| Ψ (minwater+area)p(.) | 4 | 53.98 | 38.82 | 6.20E-10 | 1 | do not converge |
| Ψ (avewater+area)p(.) | 4 | 54.29 | 39.14 | 5.30E-10 | 1 | M76 |
| Ψ (height)p(.) | 3 | 54.38 | 39.23 | 5.00E-10 | 1 | M78 |
| Ψ (height+city)p(.) | 4 | 55.02 | 39.87 | 3.70E-10 | 1 | M26 |
| Ψ (minwater)p(.) | 3 | 55.02 | 39.87 | 3.70E-10 | 1 | do not converge |
| Ψ (highways+avewater)p(.) | 4 | 55.3 | 40.15 | 3.20E-10 | 1 | M69 |
| Ψ (mining+avewater)p(.) | 4 | 55.32 | 40.16 | 3.20E-10 | 1 | M54 |
| Ψ (*Eucalyptus*+avewater)p(.) | 4 | 55.44 | 40.28 | 3.00E-10 | 1 | M47 |
| Ψ (rivers+avewater)p(.) | 4 | 55.45 | 40.29 | 3.00E-10 | 1 | M60 |
| Ψ (height+mining)p(.) | 4 | 55.68 | 40.52 | 2.60E-10 | 1 | M25 |
| Ψ (mining+minwater)p(.) | 4 | 55.85 | 40.69 | 2.40E-10 | 1 | do not converge |
| Ψ (city+area)p(.) | 4 | 55.89 | 40.74 | 2.40E-10 | 1 | M66 |
| Ψ (area)p(.) | 3 | 55.94 | 40.79 | 2.30E-10 | 1 | M88 |
| Ψ (height+*Eucalyptus*)p(.) | 4 | 56.27 | 41.12 | 2.00E-10 | 1 | M23 |
| Ψ (height+rivers)p(.) | 4 | 56.37 | 41.21 | 1.90E-10 | 1 | M25 |
| Ψ (height+highways)p(.) | 4 | 56.37 | 41.22 | 1.90E-10 | 1 | M27 |
| Ψ (rivers+minwater)p(.) | 4 | 56.39 | 41.24 | 1.80E-10 | 1 | do not converge |
| Ψ (highways+minwater)p(.) | 4 | 56.5 | 41.34 | 1.80E-10 | 1 | do not converge |
| Ψ (mining+area)p(.) | 4 | 56.62 | 41.47 | 1.60E-10 | 1 | M55 |
| Ψ (*Eucalyptus*+minwater)p(.) | 4 | 56.93 | 41.78 | 1.40E-10 | 1 | do not converge |
| Ψ (rivers+area)p(.) | 4 | 57.63 | 42.47 | 1.00E-10 | 1 | M61 |
| Ψ (*Eucalyptus*+area)p(.) | 4 | 57.68 | 42.53 | 9.70E-11 | 1 | M48 |
| Ψ (highways+area)p(.) | 4 | 57.92 | 42.77 | 8.60E-11 | 1 | M70 |
| Ψ (city)p(.) | 3 | 58.11 | 42.96 | 7.80E-11 | 1 | M83 |
| Ψ (mining)p(.) | 3 | 58.69 | 43.54 | 5.80E-11 | 1 | M81 |
| Ψ (rivers)p(.) | 3 | 59.39 | 44.23 | 4.10E-11 | 1 | M82 |
| Ψ (*Eucalyptus*)p(.) | 3 | 59.4 | 44.24 | 4.10E-11 | 1 | M80 |
| Ψ (highways)p(.) | 3 | 59.4 | 44.25 | 4.10E-11 | 1 | M84 |
| Ψ (mining+city)p(.) | 4 | 59.68 | 44.52 | 3.60E-11 | 1 | M50 |
| Ψ (city+highways)p(.) | 4 | 60.05 | 44.89 | 3.00E-11 | 1 | M62 |
| Ψ (*Eucalyptus*+city)p(.) | 4 | 60.06 | 44.91 | 3.00E-11 | 1 | M43 |
| Ψ (rivers+city)p(.) | 4 | 60.1 | 44.95 | 2.90E-11 | 1 | M56 |
| Ψ (*Eucalyptus*+mining)p(.) | 4 | 60.64 | 45.48 | 2.20E-11 | 1 | M41 |
| Ψ (mining+highways)p(.) | 4 | 60.69 | 45.53 | 2.20E-11 | 1 | M51 |
| Ψ (mining+rivers)p(.) | 4 | 60.69 | 45.54 | 2.10E-11 | 1 | M49 |
| Ψ (*Eucalyptus*+rivers)p(.) | 4 | 61.38 | 46.23 | 1.50E-11 | 1 | M42 |
| Ψ (rivers+highways)p(.) | 4 | 61.38 | 46.23 | 1.50E-11 | 1 | M57 |
| Ψ (*Eucalyptus*+highways)p(.) | 4 | 61.39 | 46.23 | 1.50E-11 | 1 | M44 |
